# Efficacy and possible mechanism of *Kai-xin-san* in animal models of Alzheimer’s disease: a systematic review and meta-analysis of preclinical studies

**DOI:** 10.3389/fphar.2026.1806181

**Published:** 2026-04-23

**Authors:** Yuzhi Wang, Qun Wang, Hui Xie, Xiaozhu Liu, Yuhuan Ju, Xifeng Liu, Shiguang Sun

**Affiliations:** 1 College of Pharmacy, Shandong University of Traditional Chinese Medicine, Jinan, China; 2 College of Traditional Chinese Medicine, Shandong University of Traditional Chinese Medicine, Jinan, China; 3 Department of Vertigo, Jinan Shizhong People’s Hospital, Jinan, China; 4 Department of Pharmacy, Second Affiliated Hospital, Shandong Provincial Hospital of Integrated Medicine, Shandong University of Traditional Chinese Medicine, Jinan, China

**Keywords:** Alzheimer’s disease, *Kai-xin-san*, traditional Chinese medicine, animal model, meta-analysis, systematic review

## Abstract

**Context:**

Alzheimer’s disease (AD) is the most common neurodegenerative disorder, which is associated with impaired cognition. *Kai-xin-san* (KXS), a classic Chinese herbal formula, has been widely used to treat cognitive disorders.

**Objectives:**

This study aimed to investigate the therapeutic potential and underlying mechanisms of KXS for AD.

**Methods:**

A systematic search of 7 databases was conducted from inception to October 2025, with language restrictions in both Chinese and English. Behavior and biomarkers were assessed as measures of efficacy and mechanism. The risk of bias was assessed by the SYRCLE’s risk of bias tool. The meta-analysis was performed by STATA version 15.0 software packages and RevMan 5.4 software. Subgroup, meta-regression, and sensitivity analyses were used to ascertain the robustness of primary analyses, Egger’s test and funnel plots were used to assess potential publication bias, and the evidence of evidence was assessed by the modified GRADE approach.

**Results:**

This study included 44 studies, involving a total of 2,681 animals, 3 behavioral tests, such as Morris water maze (MWM), novel object recognition (NOR), Y maze, and 9 biomarkers, such as β-amyloid peptide (Aβ), tau protein, tumor necrosis factor-α (TNF-α), interleukin-1β (IL-1β), and interleukin-6 (IL-6), malondialdehyde (MDA), superoxide dismutase (SOD), acetylcholinesterase (AchE), and acetylcholine (ACh). KXS could significantly shorten escape latency from the target quadrant, and elevate entry frequency into the target quadrant and time spent in the target quadrant in MWM; meanwhile, KXS could also significantly enhance the relative recognition index in NOR, and improve spontaneous alternation performance in Y-maze. Moreover, KXS could decrease Aβ, tau and AchE in the hippocampus, and TNF-α, IL-1β, IL-6, and MDA in both serum and hippocampus, while increase ACh in the hippocampus and SOD in both serum and hippocampus.

**Conclusion:**

These findings suggest that KXS may alleviates cognitive deficits in AD animal models, which may be attributed to its modulation of multiple mechanisms, including Aβ and tau pathology, inflammation, oxidative stress, and cholinergic function. However, due to the high risk of bias, indicating that the true effects of KXS may be smaller than those reported. Therefore, high-quality preclinical studies are essential before clinical efficacy can be considered.

## 1 Introduction

Alzheimer’s disease (AD) is a progressive degenerative disease of the central nervous system and the most common cause of dementia, marked by progressive cognitive decline and behavioral changes (Baviskar and Mahajan, 2026). The global number of people living with dementia is projected to increase from 57.4 million in 2019 to 152.8 million in 2050 (GBD 2019 Dementia Forecasting Collaborators, 2022). It is a major cause of illness and death, and imposes a significant economic burden on affected individuals and society (Chen S. et al., 2024). According to the current research framework, AD is diagnosed based on biomarkers, including β-amyloid peptide (Aβ) deposition, pathological tau, and neurodegeneration (Jack et al., 2018). Current treatment strategies for AD encompass both pharmacological and non-pharmacological approaches, with cholinesterase inhibitors and memantine representing the only approved antidementia drugs (Gauthier and Molinuevo, 2013; D'Angremont et al., 2023). Despite considerable initial promise, these agents have demonstrated limited efficacy and concerning adverse effects (Winslow et al., 2011). Consequently, this has prompted the exploration of alternative approaches, among which traditional Chinese medicine (TCM) has shown significant success in the prevention and treatment of various medical conditions (Chen et al., 2025).

*Kai-xin-san* (KXS), a traditional formulation first documented by *Sun Simiao*’s “*Bei-Ji-Qian-Jin-Yao-Fang*,” is a classical prescription used for amnesia, with functions including nourishing the heart, strengthening the spleen, tranquilizing the mind, and harmonizing the heart and kidneys. The formulation comprises four botanical drugs, namely, *Panax ginseng* C. A. Mey., *Polygala tenuifolia* Willd., *Poria cocos* (Schw.) Wolf, and *Acorus tatarinowii* Schott (Chen L. et al., 2024). The therapeutic effects of *P. ginseng* on AD are primarily attributed to its diverse active metabolites, most notably ginsenosides, which have been demonstrated to play a significant role in preventing and treating neurological diseases (Razgonova et al., 2019; Wang et al., 2023). *Polygala tenuifolia* exerts neuroprotective effects relevant to AD through its multiple active metabolites, particularly its saponins, which have demonstrated therapeutic potential in treating dementia (Deng et al., 2020; Li et al., 2024). *Poria cocos* contains multiple active ingredients that play a significant role in treating AD, and further research indicates that its polysaccharide metabolite specifically improves cognitive impairment (Sun et al., 2021; Song et al., 2025). *Acorus tatarinowii* Schott, whose primary metabolites include asarone and polysaccharides, has demonstrated therapeutic efficacy in the treatment of neurological disorders (Kim et al., 2022; Yu et al., 2024). Notably, modern pharmacological studies have demonstrated that the mechanism of action of KXS involves the modulation of Aβ metabolism, tau protein hyperphosphorylation, cholinergic dysfunction, and inflammatory responses (Luo et al., 2023).

A systematic review applied network pharmacology and molecular docking to investigate the multi-target mechanism of KXS against AD (Yi et al., 2020); another study demonstrated the efficacy of KXS in alleviating depressive symptoms, underscoring its potential for managing the behavioral and psychological symptoms of dementia in AD (Fu et al., 2019). However, no study has systematically and quantitatively synthesized the preclinical evidence specifically focusing on the therapeutic effects of KXS on core AD pathologies. Furthermore, no meta-analysis has comprehensively evaluated its multifaceted mechanisms, including Aβ and tau pathology, anti-inflammatory, antioxidant, and cholinergic regulation. Therefore, this study aimed to conduct a preclinical systematic review and meta-analysis to evaluate the efficacy of KXS on cognitive function in AD animal models and to comprehensively elucidate its underlying molecular mechanisms. This study will provide a robust theoretical foundation to support the clinical translation of KXS for AD treatment.

## 2 Materials and methods

This review was conducted in accordance with the Preferred Reporting Items for Systematic Reviews and Meta-Analyses (PRISMA) guidelines (Page et al., 2021) and registered with PROSPERO (CRD42024534438; CRD420251171230).

### 2.1 Search strategy

Seven databases were searched from inception to October 2025, including PubMed, Embase, Web of Science, China National Knowledge Infrastructure (CNKI), Wan Fang Database (WF), Chinese Scientific Journal Database (VIP), and Chinese Biological Medicine Database (SinoMed). The search strategy employed the terms: “*kai-xin,*” “*kai xin,*” “*kaixin,*” “Alzheimer’s disease,” “dementia,” “rodent,” as well as their respective abbreviations, and all synonymous variants for each database. The complete details are available in the Supplementary Material.

### 2.2 Inclusion criteria and exclusion criteria

The inclusion criteria were described as follows: 1) Study designs: Controlled comparative preclinical studies; 2) Participants: Animal model in AD of rodents; regardless of species, strains, age, and sex; 3) Interventions: KXS monotherapy in the experimental group; 4) Comparators: No-treatment or vehicle control (such as normal saline or distilled water); 5) Outcomes: Behaviour outcomes, such as Morris water maze (MWM) test, novel object recognition (NOR) test, etc.,; Biomarker outcomes, such as inflammation factors, oxidative stress indicators, etc.

The exclusion criteria were described as follows: 1) Clinical articles, case reports, reviews, comments, and abstracts; 2) No animal studies or with co-morbidities, *in vitro* and *ex vivo* studies; 3) Rodent models of not AD (e.g., scopolamine induced model, etc.).

### 2.3 Literature screening and data extraction

Two investigators (YZ Wang and Q Wang) independently screened the literature against the predefined inclusion and exclusion criteria. After removal of duplicates, two independent authors (YZ Wang and Q Wang) initially screened the studies by title, abstract. Subsequently, a full-text review was conducted, and the final conclusions were determined. Any matters were resolved through consensus or by adjudication from a third party (H Xie).

The extracted items included the following: 1) Study characteristics, including authors and publication year; 2) Animal characteristics, including species, sex, number, strains, age, and weight; 3) Modeling method; 4) Intervention characteristics, including intervention doses and intervention means; 5) Outcomes characteristics, including behavioral indicators and biomarker indicators. For studies reporting multiple time points or doses, data at the time point of the maximum observed effect were extracted. WebPlotDigitizer 4.5 was used to extract the mean and standard deviation from the bar plots or line charts.

### 2.4 Risk of bias

Study quality assessment was assessed by two independent reviewers (YZ Wang and Q Wang) using the Systematic Review Centre for Laboratory animal Experimentation (SYRCLE) (Hooijmans et al., 2014). Any disagreement was solved by a third reviewer (H Xie). SYRCLE’s risk of bias assessment method for animal experiments included 1) Random sequence generation; 2) Baseline characteristics; 3) Allocation concealment; 4) Random housing; 5) Blinding of participants and personnel; 6) Random outcome assessment; 7) Blinding of outcome assessment; 8) Incomplete outcome data; 9) Selective reporting; 10) Other bias. Each entry was assessed as “low risk,” “unclear risk,” or “high risk.”

### 2.5 Statistical analysis

This meta-analysis performed the data analysis using RevMan 5.4 and Stata 15.0, and the standardized mean difference (SMD; Hedge’s g) with 95% confidence interval (95% CI) was used for continuous data to pool the results. This meta-analysis assessed statistical heterogeneity in each pairwise comparison with the *I* ^*2*^ statistic and *P* value (Higgins and Thompson, 2002). A random-effects model was used in the significantly statistical heterogeneity (*I* ^*2*^ > 50%). In cases of substantial heterogeneity, subgroup meta-analysis and meta-regression were conducted to identify a potential source. Sensitivity analysis was performed to evaluate the robustness of the pooled results. For outcomes involving more than 10 studies, publication bias was assessed visually with funnel plots and statistically using Egger’s test and applied the trim-and-fill method.

### 2.6 GRADE certainty of the evidence

The certainty of evidence was assessed using a modified Grading of Recommendations Assessment Development and Evaluation (GRADE) approach developed for systematic reviews of preclinical studies (Hooijmans et al., 2018). This assessment tool comprises five domains, including the risk of bias, indirectness, inconsistency, imprecision, and publication bias. A summary of the GRADE certainty of evidence was generated using the free online software GRADEpro.

## 3 Results

### 3.1 Search results

**FIGURE 1:**
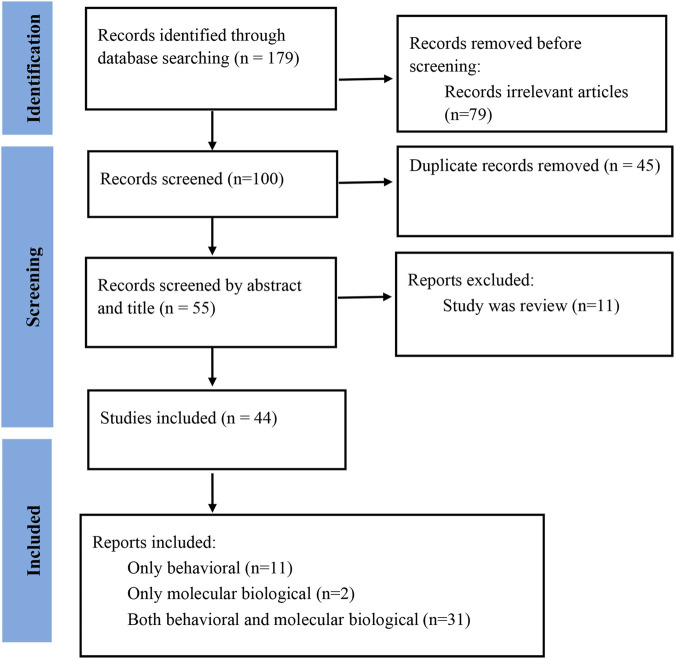
The flowchart of paper inclusion process.

A total of 179 studies were collected through the search strategy. This study did not restrict studies by publication year, and relevant studies published between 1999 and 2025 were identified. After removing irrelevant and duplicate studies, 55 records were retained. Two investigators (YZ Wang and Q Wang) independently conducted the literature screening based on specified inclusion and exclusion criteria. Finally, 44 studies (Huang et al., 1999; Zhong, 2005; Zhou et al., 2008; Gao et al., 2010; Shi et al., 2013; Lv, 2015; Yu, 2015; Chu et al., 2016; Li et al., 2016; Wang, 2016; Chai, 2017; Han et al., 2017; Shi et al., 2017a; Shi et al., 2017b; Xu, 2017; Liu, 2018; Wang, 2018; Du, 2019; Guo et al., 2019; Li et al., 2020; Liu et al., 2021; Wang et al., 2021; Jiao et al., 2022; Liang, 2022; Lou et al., 2022; Pei et al., 2022; Li M., 2023; Li R., 2023; Li S., 2023; Lu, 2023; Su et al., 2023; Xu et al., 2023; Chen L. et al., 2024; Liu, 2024; Shan et al., 2024a; Shan et al., 2024b; Wang, 2024; Yan et al., 2024; Cai et al., 2025; Kang et al., 2025; Li et al., 2025; Ma et al., 2025; Yang et al., 2025; Zhou et al., 2025) were included in the final analysis (Figure 1).

### 3.2 Study characteristics

The analysis included 44 studies, encompassing a total of 2,681 animals. In terms of species, 23 studies used transgenic mice, of which 15 studies used APP/PS1 mice, 3 studies used 5×FAD mice, 5 studies used SAMP8 mice. Sprague Dawley (SD) rats were used in 10 studies, with intraperitoneal injection of D-galactose (D-gal) in 3 studies, hippocampal injection of Aβ_1-42_ in 2 studies, intracerebroventricular injection of streptozotocin (STZ) in 1 study and mixed injection of D-gal + Aβ_25-35_ in 4 studies. Wistar rats were used in 4 studies, with hippocampal injection of Aβ_1-42_ in 1 study, hippocampal injection of Aβ_25-35_ in 1 study, and mixed injection of D-gal + Alcl_3_ in 2 studies. ICR mice were used in 2 studies, with hippocampal injection of Aβ_1-42_ in 1 study, and administering Alcl_3_ in 1 study. Kun Ming (KM) mice were used in 5 studies, with intraperitoneal injection of D-gal in 2 studies, and mixed injection of D-gal + Alcl_3_ and D-gal + Nano_2_ in 3 studies.

In terms of behavioral indicators, 39 studies performed the MWM test. In these studies, 36 studies reported escape latency, 35 studies reported entry frequency into the target quadrant, 17 studies reported crossing distance in the target quadrant, and 26 studies reported time spent in the target quadrant. 10 studies performed the NOR test and 12 studies performed the Y-Maze test. In terms of molecular biology indicators, 44 studies included indicators of inflammatory factors, of which tumor necrosis factor-α (TNF-α) was involved in 18 studies, nterleukin-1β (IL-1β) was involved in 19 studies, interleukin-6 (IL-6) was involved in 13 studies; 20 studies included oxidative stress indicators, of which 9 studies involved malondialdehyde (MDA), 11 studies involved superoxide dismutase (SOD); 20 studies included cholinergic system indicators, of which 6 studies involved acetylcholine (ACh), 9 studies involved acetyl cholinesterase (AchE); 14 studies included Aβ and tau protein factors, of which 9 studies involved Aβ, 5 involved tau protein, and the complete details are available in Table 1.

**TABLE 1:** The main characteristics of the included studies.

Study	Animal				Model	Intervention			Outcome	
	Species	Strains	Sex	Weight		Formulation	Dose	Administration route/Frequency/Duration	Behavioral outcomes	Molecular biological outcomes
Liu et al. (2021)	Rat	SD	Male	200–240 g	Aβ_1-42_	KXS Decoction	40 g/kg	i.g. q.d. 28 d	MWM	SOD
Wang (2016)	Mouse	APP/PS1	Male	28–32 g	APP/PS1	KXS Extraction	3 g/kg	i.g. q.d. 122 d	MWM	Aβ Tau
Luo et al. (2022)	Mouse	ICR	Male	22–25 g	Aβ_1-42_	KXS Extraction	10 g/kg	NR q.d. 7 d	MWM Y-maze	TNF-α IL-1β IL-6
Lv (2015)	Rat	SD	Male	230–270 g	D-gal	KXS Extraction	9.4 g/kg	i.g. q.d. 35 d	MWM	SOD MDA ChE
Chu et al. (2016)	Rat	Wistar	Male	240–280 g	AlCl_3_, D-gal	KXS Extraction	5.4 g/kg	i.g. q.d. 90 d	MWM	\
Wang (2018)	Rat	SD	Male	220–250 g	D-gal, Aβ_25-35_	KXS Extraction	10 g/kg	i.g. q.d. 35 d	MWM	\
Liu (2018)	Mouse	5×FAD	Both	22–30 g	5×FAD	KXS Decoction	8.6 g/kg	i.g. q.d. 90 d	MWM NOR Y-maze	\
Du (2019)	Mouse	5×FAD	Both	20–30 g	5×FAD	KXS Decoction	8.7 g/kg	i.g. q.d. 90 d	MWM NOR Y-maze Open field	\
Liang (2022)	Mouse	5×FAD	Both	22–30 g	5×FAD	KXS Extraction	8.6 g/kg	i.g. q.d. 90 d	NOR Y-maze Open field	IL-6 Aβ
Li M. (2023)	Rat	APP/PS1	Male	25–30 g	APP/PS1	KXS Extraction	10 g/kg	i.g. q.d. 28 d	MWM NOR Open field	SOD MDA
Li R. (2023)	Mouse	SAMP8	Male	35–40 g	SAMP8	KXS Extraction	2.8 g/kg	i.g. q.d. 28 d	MWM NOR Open field	\
Cheng et al. (2024)	Mouse	KM	Male	28–32 g	AlCl_3_, D-gal	KXS Decoction	5.4 g/kg	i.g. b.i.d. 90 d	MWM	IL-1β IL-18
Huang et al. (1999)	Mouse	ICR	Male	17–20 g	AlCl_3_	KXS Extraction	0.77 g/kg	i.g. q.d. 90 d	MWM	SOD MDA
Zhong (2005)	Rat	Wistar	Both	400–450 g	Aβ_25-35_	KXS Extraction	0.3 g/kg	i.g. q.d. 28 d	MWM	AchE
Gao et al. (2010)	Mouse	KM	Male	28–30 g	D-gal	KXS Decoction	10 g/kg	i.g. NR NR	MWM	AchE SOD MDA
Shi et al. (2013)	Mouse	SAMP8	Male	27.2–31.52 g	SAMP8	KXS Decoction	39 g/kg	i.g. NR 56 d	\	TNF-α IL-8
Li et al. (2016)	Mouse	KM	Both	18–22 g	D-gal, Nano_2_	KXS Granule	3.57 g/kg	i.g. q.d. 35 d	MWM	AchE Ach Aβ Tau
Xu (2017)	Rat	Wistar	NR	250–300 g	Aβ_1-42_	KXS Decoction	5.6 g/kg	i.g. q.d. 28 d	MWM	\
Shi et al. (2017a)	Mouse	SAMP8	Male	24.5–34.2 g	SAMP8	KXS Decoction	39 g/kg	i.g. q.d. 64 d	\	NE
Han et al. (2017)	Mouse	APP/PS1	Male	21–25 g	APP/PS1	KXS Decoction	7.8 g/kg	i.g. NR 30 d	MWM	TNF-α IL-1β IL-6
Chai (2017)	Mouse	APP/PS1	Both	18–22 g	APP/PS1	KXS Extraction	2 g/kg	i.g. q.d. 180 d	MWM	AchE ACH ChAT Aβ
Li et al. (2020)	Mouse	KM	Both	18–22 g	AlCl_3_, D-gal	KXS Extraction	48 g/kg	i.g. q.d. 28 d	MWM	\
Wang et al. (2021)	Mouse	APP/PS1	Male	20–30 g	APP/PS1	KXS Extraction	3.00 g/kg	i.g. q.d. 60 d	MWM	TNF-α IL-1β IL-6 Ach Aβ
Pei et al. (2022)	Rat	SD	Both	260–300 g	Aβ_1-42_	KXS Extraction	0.04592 g/kg	i.g. q.d. 30 d	MWM	TNF-α IL-1β IL-6 AchE ACh CHAT Aβ Tau
Lu (2023)	Mouse	APP/PS1	Male	26–30 g	APP/PS1	KXS Extraction	2.8 g/kg	i.g. q.d. 60 d	Y-maze test	TNF-α IL-1β IL-6
Wang (2024)	Rat	SD	Male	180–220 g	D-gal, Aβ_25-35_	KXS Extraction	10 g/kg	i.g. q.d. 42 d	MWM	TNF-α IL-1β IL-6
Kang et al. (2025)	Rat	SD	Male	180–220 g	STZ	KXS Granule	3.08 g/kg	i.g. q.d. 30 d	MWM NOR Y-maze	SOD
Yang et al. (2025)	Mouse	APP/PS1	Male	20–24 g	APP/PS1	KXS Granule	6.5 g/kg	i.g. b.i.d. 168 d	MWM NOR Y-maze	TNF-α IL-1β IL-6
Shi et al. (2017b)	Mouse	SAMP8	Male	24.5–34.2 g	SAMP8	KXS Decoction	39 g/kg	i.g. q.d. 56 d	MWM	\
Zhou et al. (2008)	Mouse	KM	Male	18–22 g	D-gal	KXS Extraction	0.9 g/kg	i.g. q.d. NR	MWM	SOD MDA
Cai et al. (2025)	Rat	SD	Male	180–220 g	D-gal	KXS Extraction	0.4 g/kg	i.g. q.d. 21 d	MWM	TNF-α IL-1β IL-6
Guo et al. (2019)	Rat	SD	Male	200–250 g	D-gal	KXS Extraction	10 g/kg	i.g. q.d. 7 d	MWM	TNF-α IL-1β Tau
Zhou et al. (2025)	Rat	SD	Male	180–220 g	D-gal, Aβ_25-35_	KXS Extraction	10 g/kg	i.g. b.i.d. 28 d	MWM	TNF-α IL-1β IL-6
Shan et al. (2024a)	Rat	SD	NR	NR	D-gal, Aβ_25-35_	KXS Extraction	4 g/kg	i.g. q.d. 28 d	MWM	TNF-α IL-1β SOD MDA ACh
Jiao et al. (2022)	Mouse	SAMP8	Male	28–32 g	SAMP8	KXS Decoction	5.4 g/kg	i.g. q.d. 90 d	MWM	TNF-α IL-1β IL-6 Tau
Su et al. (2023)	Mouse	APP/PS1	Male	NR	APP/PS1	KXS Extraction	10 g/kg	i.g. q.d. 30 d	MWM NOR	IL-1β IL-18 SOD MDA
Shan et al. (2024b)	Mouse	APP/PS1	NR	NR	APP/PS1	KXS Extraction	1.0569 g/kg	i.g. NR NR	MWM NOR	TNF-α IL-1β IL-6 IL-18 AchE MOD MDA
Yan et al. (2024)	Mouse	APP/PS1	Male	25–33 g	APP/PS1	KXS Decoction	2.8 g/kg	i.g. q.d. 56 d	MWM Y-maze	\
Li et al. (2025)	Mouse	APP/PS1	NR	NR	APP/PS1	KXS Extraction	1.0569 g/kg	i.g. q.d. 28 d	MWM Y-maze	TNF-α IL-1β IL-6 AchE MOD MDA Aβ
Li S. (2023)	Mouse	APP/PS1	Male	NR	APP/PS1	KXS Extraction	3.0 g/kg	i.g. q.d. 60 d	MWM	TNF-α IL-1β
Yu (2015)	Rat	Wistar	Male	240–280 g	D-gal, AlCl_3_	KXS Extraction	7.2 g/kg	i.g. q.d. 105 d	MWM	Aβ
Liu (2024)	Mouse	APP/PS1	NR	18–22 g	APP/PS1	KXS Granule	6.5 g/kg	i.g. b.i.d. 36 d	MWM	ACh AchE
Ma et al. (2025)	Mouse	APP/PS1	Male	25–33 g	APP/PS1	KXS Extraction	2.8 g/kg	i.g. q.d. 56 d	MWM Y-maze	TNF-α IL-1β IL-6 Aβ
Xu et al. (2023)	Mouse	APP/PS1	Male	NR	APP/PS1	KXS Decoction	2.8 g/kg	i.g. q.d. 60 d	NOR Y-maze	TNF-α IL-1β IL-6

MWM, morris water maze; NOR, novel object recognition; TNF-α, tumor necrosis factor-α; IL-1β, interleukin-1β; IL-6, interleukin-6; IL-8, interleukin-8; IL-18, interleukin-18; MDA, malondialdehyde; SOD, superoxide dismutase; ACh, acetylcholine; AchE, acetyl cholinesterase; ChAT, choline acetyl cholinesterase; ChE, cholinesterase; NE, norepinephrine; D-gal, D-galactose; STZ, streptozocin; q.d., quaque die; b.i.d., bis in die; i.g., intragastris; KXS, *Kai-xin-san*; Aβ, β-amyloid peptide; NR: not reported.

### 3.3 Risk of bias

**FIGURE 2:**
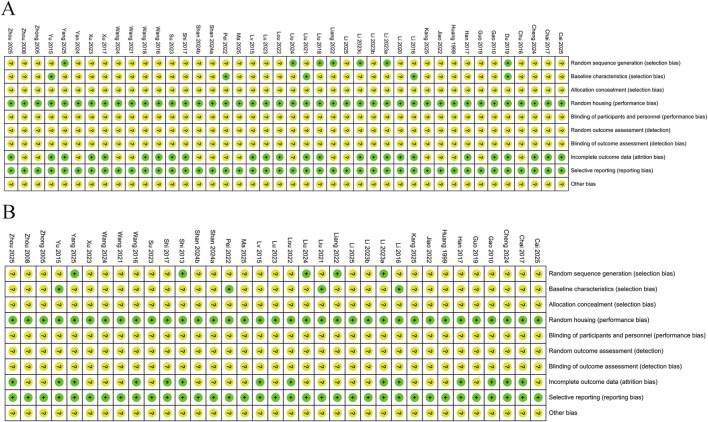
The quality assessment of the risk of bias. **(A)** Behavioral outcomes; **(B)** Molecular biology outcomes.

Regarding behavioral indicators, this study conducted a quality assessment of the risk of bias methodology in 42 studies. Among these, 7 studies described their randomization method in detail, which this was not specified in the remaining 35. Similarly, missing data were unclear in 16 studies, and were explicitly reported as complete in the remaining 26 studies, and 5 studies were rated as low risk in baseline characteristics. Regarding biomarker outcomes, indicators, this study conducted a quality assessment of the risk of bias methodology in 35 studies. Among these, 5 studies described their randomization method in detail; missing data were unclear in 21 studies, and 4 studies were rated as low risk in baseline characteristics. Overall, the risk of bias assessment indicates that the majority of the included studies suffer from poor methodological reporting. The high proportion of unclear risks particularly in key areas such as allocation concealment and baseline characteristics creates substantial uncertainty about the validity of individual study findings, and this limitation is critical. The complete details are available in Figure 2.

### 3.4 Effects of KXS on behavioral performance in AD

**FIGURE 3:**
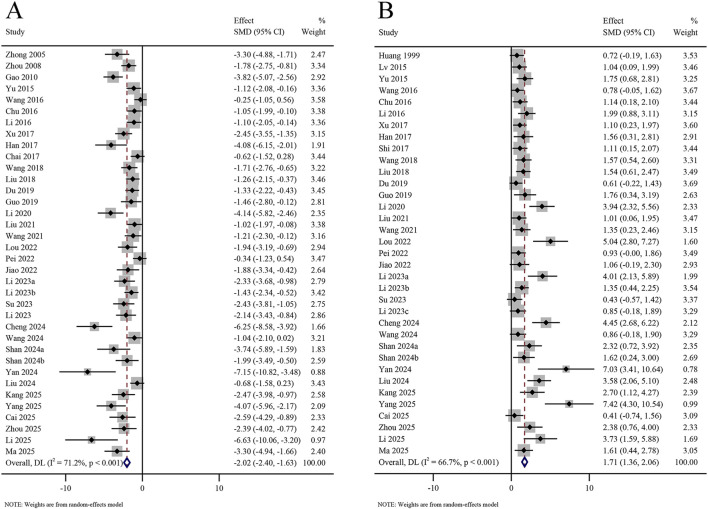
Forest plot for the effect of *Kai-xin-san* on the Morris water maze test (I). **(A)** Escape latency from the target quadrant; **(B)** Entry frequency into the target quadrant.

A total of 36 studies reported on escape latency in the MWM test, involving 2,293 animals. The meta-analysis revealed that escape latency was significantly shorter in the KXS group than in the model group (SMD = −2.02, 95% CI [−2.40, −1.63]), with certain heterogeneity (*I* ^*2*^ = 71.2%, *P* < 0.001) (Figure 3A). A total of 35 studies reported entry frequency into the target quadrant in the MWM test, involving 2,241 animals. The meta-analysis revealed that entry frequency into the target quadrant was significantly elevated in the KXS group compared to the model group (SMD = 1.71, 95% CI [1.36, 2.06]), with certain heterogeneity (*I* ^*2*^ = 66.7%, *P* < 0.001) (Figure 3B).

**FIGURE 4:**
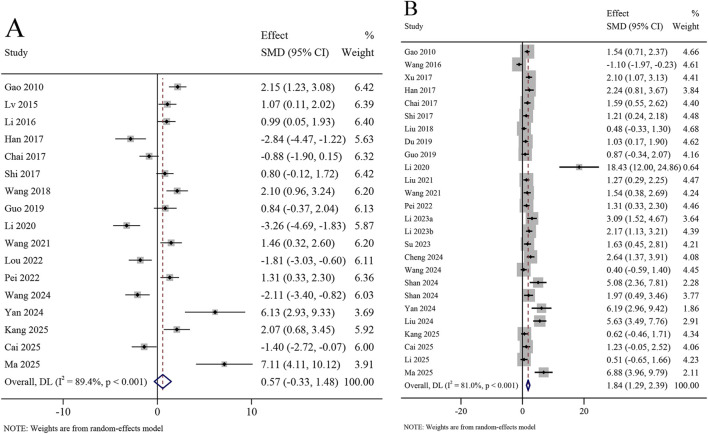
Forest plot for the effect of *Kai-xin-san* on the Morris water maze test (II). **(A)** Crossing distance in the target quadrant; **(B)** Time spent in the target quadrant.

A total of 17 studies reported the crossing distance in the target quadrant in the MWM test, involving 1,206 animals. The meta-analysis revealed that the crossing distance in target quadrant did not increase significantly in the KXS group compared to the model group (SMD = 0.57, 95% CI [−0.33, 1.48]; *I* ^*2*^ = 89.4%, *P* < 0.001) (Figure 4A). A total of 26 studies reported the time spent in the target quadrant in the MWM test, involving 1,545 animals. The meta-analysis revealed that the time spent in the target quadrant was significantly increased in the KXS group compared to the model group (SMD = 1.84, 95% CI [1.29, 2.39]), with certain heterogeneity (*I* ^*2*^ = 81.0%, *P* < 0.001) (Figure 4B).

**FIGURE 5:**
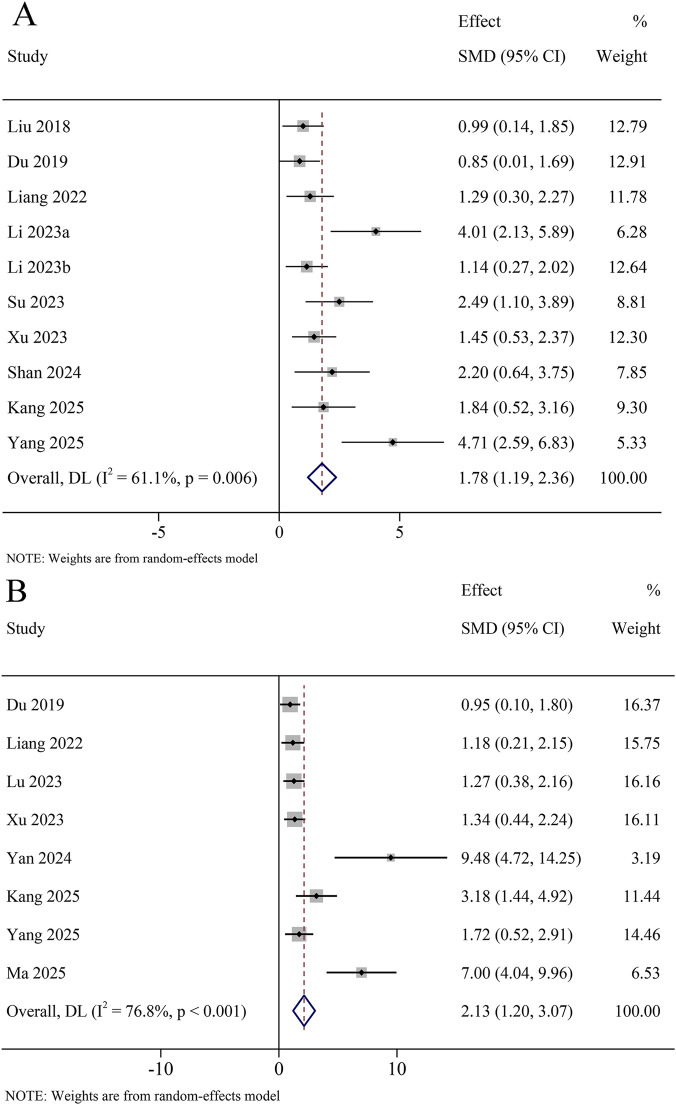
Forest plot for the effect of *Kai-xin-san* on the novel object recognition and Y-maze test. **(A)** Relative identification index in the novel object recognition test; **(B)** Spontaneous alternation performance in the Y-maze test.

A total of 26 studies reported the NOR test, involving 580 animals. The meta-analysis revealed that the relative recognition index was significantly increased in the KXS group compared to the model group (SMD = 1.78, 95% CI [1.19, 2.36]), with certain heterogeneity (*I* ^*2*^ = 61.1%, *P* = 0.006) (Figure 5A). A total of 8 studies reported the Y-maze test, involving 446 animals. The meta-analysis revealed that the spontaneous alternation performance was significantly increased in the KXS group compared to the model group (SMD = 2.13, 95% CI [1.20, 3.07]), with certain heterogeneity (*I* ^*2*^ = 76.8%, *P* < 0.001) (Figure 5B).

### 3.5 Effects of KXS on biomarkers in AD

#### 3.5.1 Amyloid and tau protein system

**FIGURE 6:**
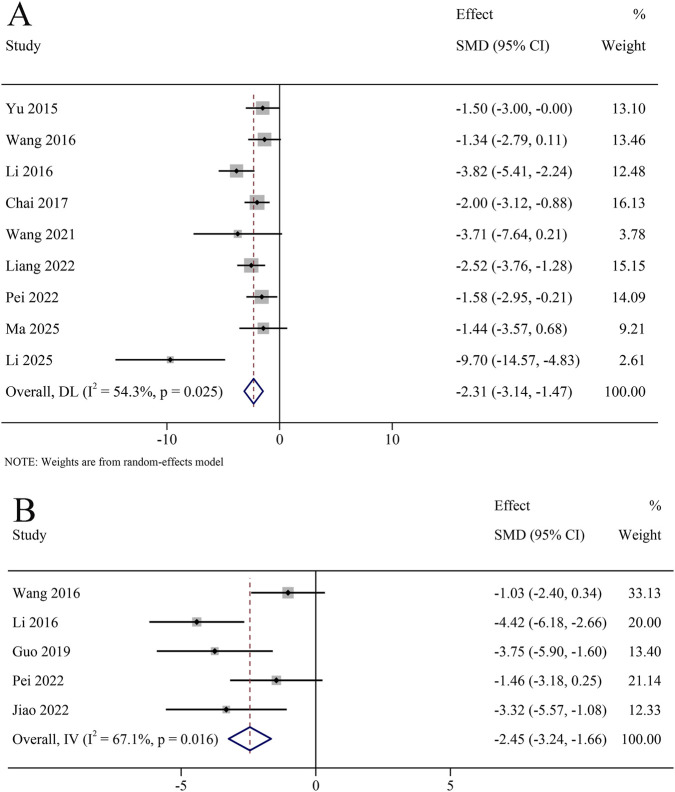
Forest plot for the effect of *Kai-xin-san* on the amyloid and tau protein system in the hippocampus **(A)** β-amyloid peptide; **(B)** tau levels.

A total of 9 studies reported Aβ in the hippocampus, involving 116 animals. The results revealed that Aβ content was significantly decreased in the KXS group compared to the model group (SMD = −2.31, 95% CI [−3.14, −1.47]), with certain heterogeneity (*I* ^*2*^ = 54.3%, *P* = 0.025) (Figure 6A).

A total of 5 studies reported tau in the hippocampus, involving 60 animals. The results revealed that tau content was significantly decreased in the KXS group compared to the model group (SMD = −2.69, 95% CI [−4.11, −1.28]), with certain heterogeneity (*I* ^*2*^ = 67.1%, *P* = 0.016) (Figure 6B).

#### 3.5.2 Neuroinflammatory system

**FIGURE 7:**
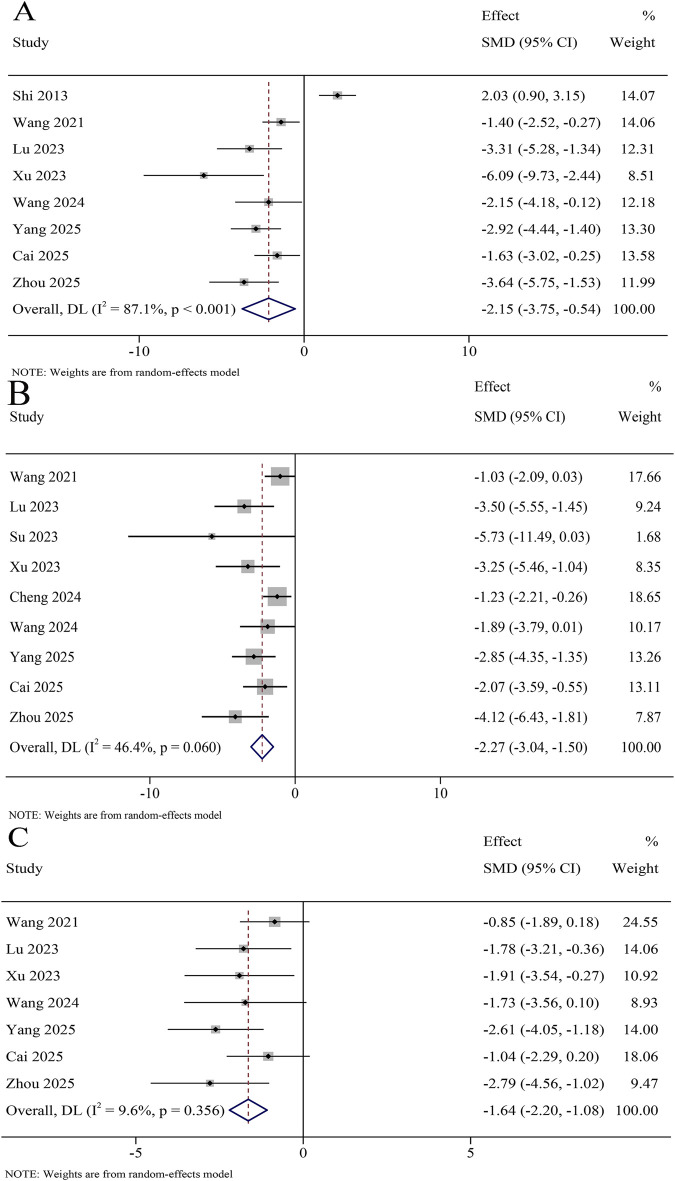
Forest plot for the effect of *Kai-xin-san* on inflammatory factors in the serum. **(A)** Tumor necrosis factor-α levels; **(B)** Interleukin-1β levels; **(C)** Interleukin-6 levels.

**FIGURE 8:**
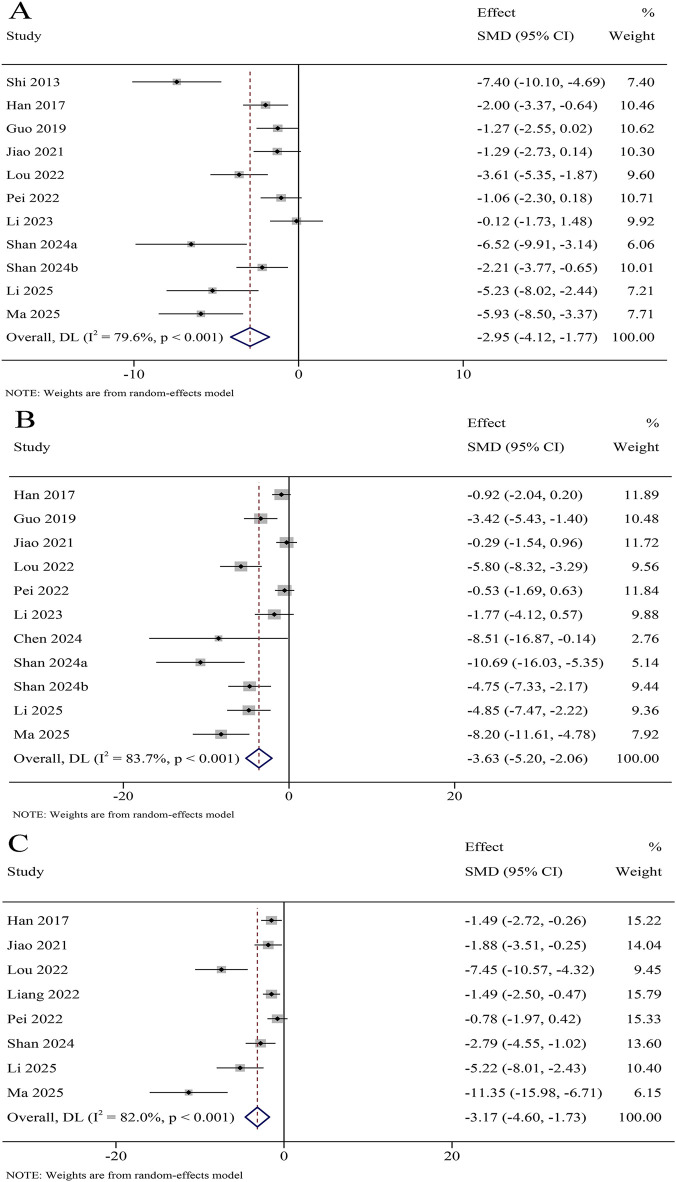
Forest plot for the effect of *Kai-xin-san* on inflammatory factors in the hippocampus. **(A)** Tumor necrosis factor-α levels; **(B)** Interleukin-1β levels; **(C)** Interleukin-6 levels.

A total of 8 studies reported TNF-α levels in the serum, involving 366 animals. The results revealed that TNF-α levels were significantly decreased in the KXS group compared to the model group (SMD = −2.15, 95% CI [−3.75, −0.54]), with certain heterogeneity (*I* ^*2*^ = 87.1%, *P* < 0.001) (Figure 7A). A total of 11 studies reported TNF-α levels in the hippocampus, involving 546 animals. The results revealed that TNF-α levels were significantly decreased in the KXS group compared to the model group (SMD = −2.95, 95% CI [−4.12, −1.77]), with certain heterogeneity (*I* ^*2*^ = 79.6%, *P* < 0.001) (Figure 8A).

A total of 9 studies reported IL-1β levels in the serum, involving 404 animals. The results revealed that IL-1β levels were significantly decreased in the KXS group compared to the model group (SMD = −2.27, 95% CI [−3.04, −1.50]), with low heterogeneity (*I^2^* = 46.4%, *P* = 0.060) (Figure 7B). A total of 11 studies reported IL-1β levels in the hippocampus, involving 536 animals. The results revealed that IL-1β levels were significantly decreased in the KXS group compared to the model group (SMD = −3.63, 95% CI [−5.20, −2.06]), with certain heterogeneity (*I^2^* = 83.7%, *P* < 0.001) (Figure 8B).

A total of 7 studies reported IL-6 levels in the serum, involving 316 animals. The results revealed that IL-6 levels were significantly decreased in the KXS group compared to the model group (SMD = −1.64, 95% CI [−2.20, −1.08]), with low heterogeneity (*I* ^*2*^ = 9.6%, *P* = 0.0356) (Figure 7C). A total of 8 studies reported IL-6 levels in the hippocampus, involving 346 animals. The results revealed that IL-6 levels were significantly decreased in the KXS group compared to the model group (SMD = −3.17, 95% CI [−4.60, −1.73]), with certain heterogeneity (*I* ^*2*^ = 82.0%, *P* = 0.001) (Figure 8C).

#### 3.5.3 Oxidative stress system

**FIGURE 9:**
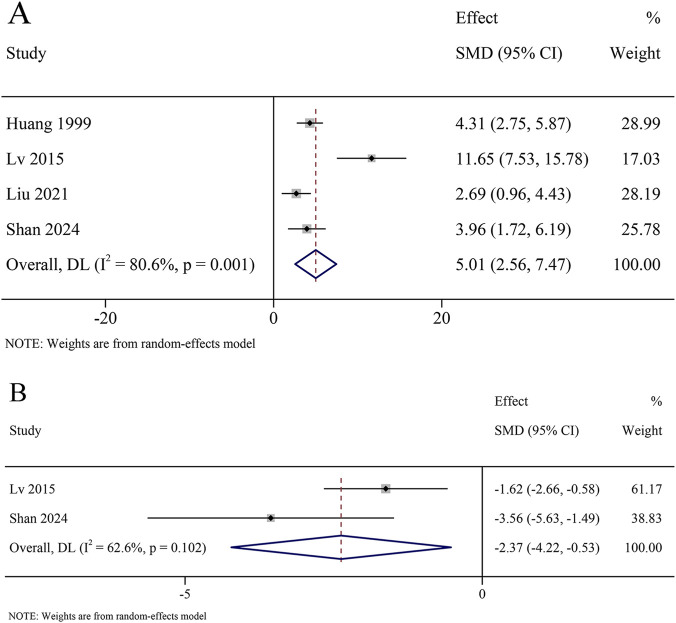
Forest plot for the effect of *Kai-xin-san* on oxidative stress factors in the serum. **(A)** Superoxide dismutase activity; **(B)** Malondialdehyde levels.

**FIGURE 10:**
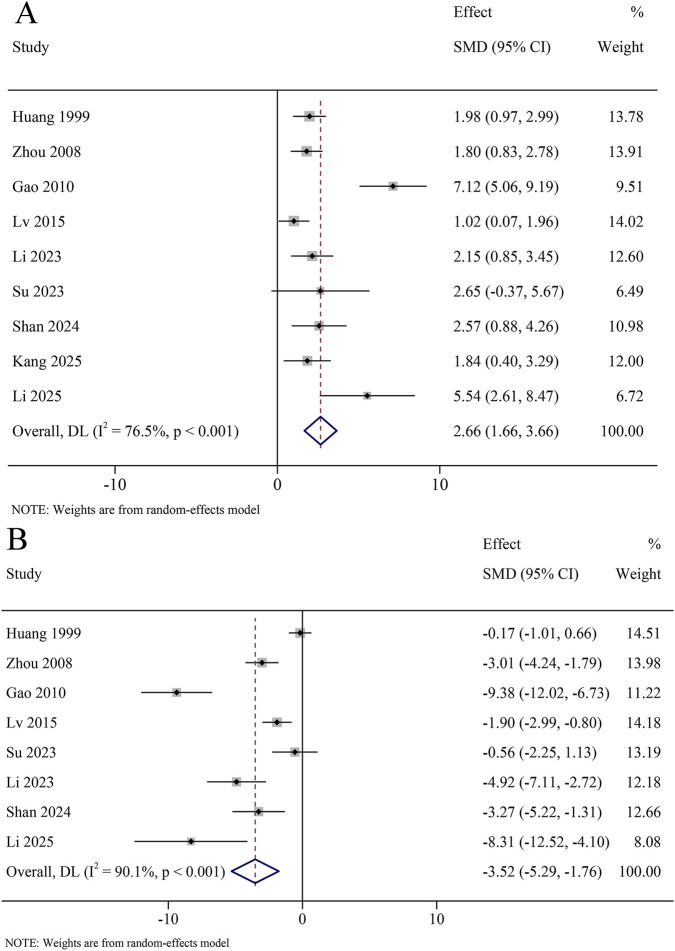
Forest plot for the effect of *Kai-xin-san* on oxidative stress factors in the hippocampus. **(A)** Superoxide dismutase activity; **(B)** Malondialdehyde levels.

A total of 4 studies reported SOD activity in the serum, involving 238 animals. The results revealed that SOD activity was significantly increased in the KXS group compared to the model group [SMD = 5.01, 95% CI (2.56, 7.47)], with certain heterogeneity (*I* ^*2*^ = 80.6%, *P* = 0.001) (Figure 9A). A total of 9 studies reported SOD content in the hippocampus, involving 466 animals. The results revealed that SOD content was significantly increased in the KXS group compared to the model group (SMD = 2.66, 95% CI [1.66, 3.66]), with certain heterogeneity (*I* ^*2*^ = 76.5%, *P* < 0.001) (Figure 10A).

A total of 2 studies reported MDA levels in the serum, involving 96 animals. The results revealed that the MDA levels were significantly decreased in the KXS group compared to the model group (SMD = −2.37, 95% CI [−4.22, −0.53]), with certain heterogeneity (*I* ^*2*^ = 62.6%, *P* = 0.102) (Figure 9B). A total of 8 studies reported MDA levels in the hippocampus, involving 406 animals. The results revealed that the MDA levels were significantly decreased in the KXS group than in the model group (SMD = −3.52, 95% CI [−5.29, −1.76]), with certain heterogeneity (*I* ^*2*^ = 90.1%, *P* < 0.001) (Figure 10B).

#### 3.5.4 Cholinergic system

**FIGURE 11:**
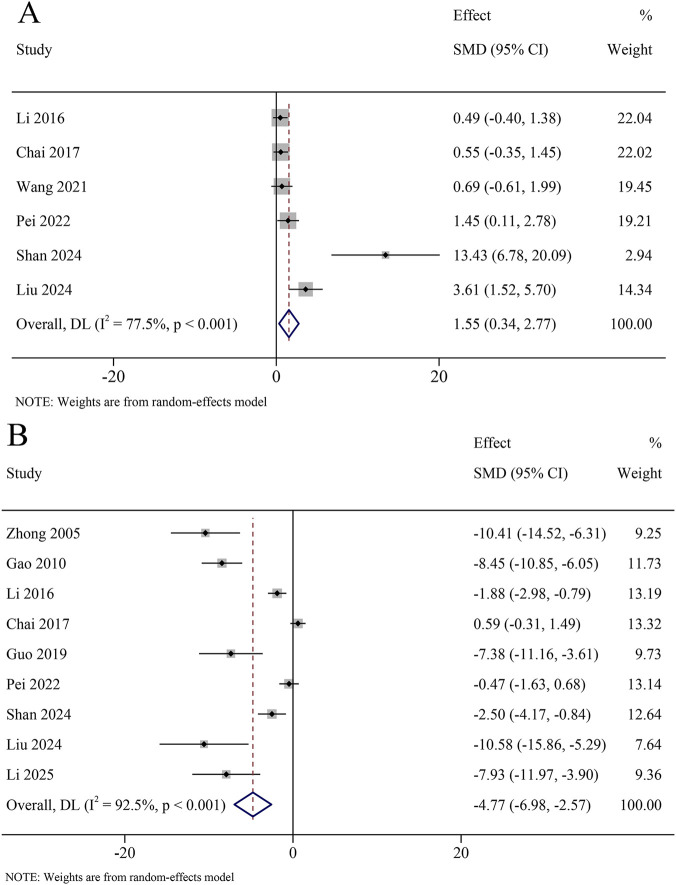
Forest plot for the effect of *Kai-xin-san* on the cholinergic system in the hippocampus **(A)** Acetylcholine content; **(B)** Acetyl cholinesterase levels.

A total of 6 studies reported ACh content in the hippocampus, involving 481 animals. The results revealed that ACh content was significantly increased in the KXS group compared to the model group (SMD = 1.55, 95% CI [0.34, 2.77]), with certain heterogeneity (*I* ^*2*^ = 77.5%, *P* < 0.001) (Figure 11A).

A total of 9 studies reported AchE levels in the hippocampus, involving 663 animals. The results revealed that AchE levels were significantly decreased in the KXS group compared to the model group (SMD = −4.77, 95% CI [−6.98, −2.57]), with certain heterogeneity (*I* ^*2*^ = 92.5%, *P* < 0.001) (Figure 11B).

### 3.6 Subgroup meta-analysis and meta-regression

We further performed subgroup meta-analysis and meta-regression according to modeling methods, species, and strains; however, the heterogeneity remained unchanged (*P >* 0.05). Notably, subgroup analysis demonstrated that animal strains were a significant source of heterogeneity in tau protein (*P* = 0.023) (Supplementary Tables S1,S2). To explore the sources of heterogeneity, we performed subgroup analyses according to the risk of bias in baseline characteristics. The results demonstrated that the differences between subgroups were statistically significant, including escape latency from the target quadrant (*P* = 0.000), entry frequency into the target quadrant (*P* = 0.042), NOR (*P* = 0.046), Y-maze (*P* = 0.035), as well as hippocampal levels of TNF-α (*P* = 0.020), IL-1β (*P* = 0.001), IL-6 (*P* = 0.005), and AchE (*P* = 0.010) (Supplementary Table S3).

### 3.7 Sensitivity analysis and publication bias

**FIGURE 12:**
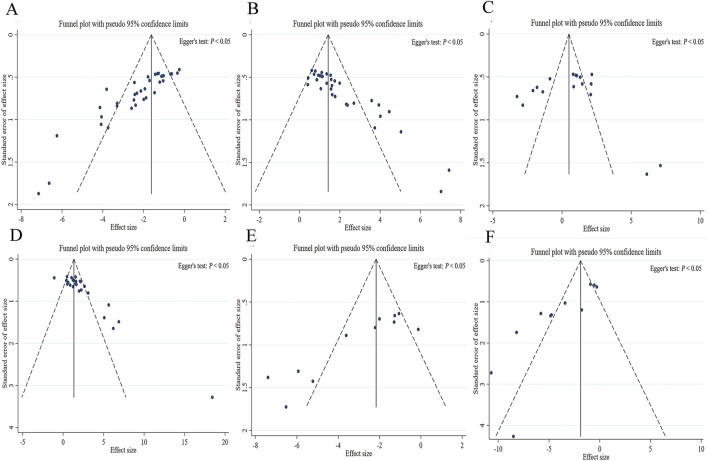
Publication bias for the effect of *Kai-xin-san*. **(A)** Escape latency from the target quadrant; **(B)** Entry frequency into the target quadrant; **(C)** Time spent in the target quadrant; **(D)** Crossing distance in the target quadrant; **(E)** Tumor necrosis factor-α levels in the hippocampus; **(F)** interleukin-1β levels in the hippocampus.

Sensitivity analyses were conducted to evaluate the influence of individual studies on the overall results. The findings demonstrated that no single study significantly altered the outcomes, indicating the robustness of results (Supplementary Figure S1). The risk of publication bias was assessed using funnel plots and Egger’s test. The funnel plots exhibited marked asymmetry, suggesting potential publication bias. At the same time, Egger’s test was assessed, which confirmed significant publication bias (Egger’s, *P* < 0.05) (Figure 12). Despite the existence of publication bias, the trim-and-fill analysis demonstrated that the overall conclusion is robust (Supplementary Figure S2).

### 3.8 GRADE certainty of the evidence

Based on the GRADE criteria, the certainty of evidence was considered as low or very low for most outcomes, with the exception of tau protein indicators, which were assessed as moderate. The detailed GRADE certainty assessment was presented in Supplementary Table S4.

### 3.9 Potential mechanisms of KXS in AD

**FIGURE 13:**
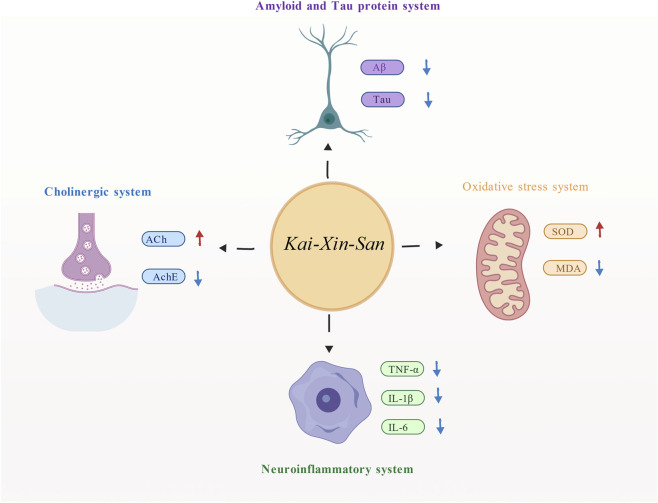
The potential mechanisms of *Kai-xin-san* in Alzheimer’s disease animal models. Notes: Aβ, β-amyloid peptide; MDA, malondialdehyde; SOD, superoxide dismutase; TNF-α, tumor necrosis factor-α; IL-1β, interleukin-1β; IL-6, interleukin-6; AchE, acetyl cholinesterase; Ach, acetylcholine. Created with BioGDP.com (Jiang et al., 2025).

The possible mechanisms of KXS concluded from the included studies are illustrated in Figure 13 and summarized in Table 2. The mechanisms are primarily multifaceted, including anti-inflammatory effects, antioxidant activity, regulation of the cholinergic system, and modulation of Aβ pathology and tau phosphorylation.

**TABLE 2:** Possible mechanisms of *Kai-xin-san* for Alzheimer’s disease**.**

Study	Regions	Potential mechanisms	Molecular changes
Huang et al. (1999)	Serum, brain	Oxidative stress system	↑SOD, ↓MDA, P < 0.05
Zhong (2005)	Brain	1. Cholinergic system 2. Mitochondrial system	1. ↓AchE, P < 0.05 2. ↓Bax, ↑Bcl-2, P < 0.05
Zhou et al. (2008)	Brain	Oxidative stress system	↑SOD, ↓MDA, P < 0.05
Gao et al. (2010)	Brain	1. Oxidative stress system 2. Cholinergic system	1. ↑SOD, ↓MDA, P < 0.01 2. ↓AchE, P < 0.01
Shi et al. (2013)	Serum, brain	1. Neuroinflammatory system 2. Amyloid protein system	1. ↓IL-8, IL-1β, P < 0.01 2. ↓β-APP, P < 0.01
Lv (2015)	Serum, brain	1. Oxidative stress system 2. Cholinergic system	1. ↑SOD, ↓MDA, P < 0.01 2. ↓ChE, P < 0.01
Yu (2015)	Brain	Amyloid protein system	↓Aβ_1-40_, P < 0.05
Li et al. (2016)	Brain	1. Neurotrophic factor system 2. Cholinergic system 3. Amyloid protein system 4. Tau pathological system	1. ↑BDNF, P < 0.01 2. ↑Ach, ↓AchE, P < 0.05 3. ↓Aβ, P < 0.05 4. ↓Tau, p-Tau, P < 0.01
Wang (2016)	Brain	1. Tau pathological system 2. Amyloid protein system	1. ↓Tau, p-Tau, P < 0.01 2. ↓Aβ, P < 0.01
Xu (2017)	Brain	Mitochondrial system	↑Bcl-2, Caspase-3, ↓Bax, P < 0.05, P < 0.01
Shi et al. (2017a)	Brain	Monoamine neurotransmitter system	↑5-HT, 5-HIAA, NE, DA, P < 0.01
Shi et al. (2017b)	Brain	Mitochondrial system	↑Bcl-2, mtDNA, ↓Bax, P < 0.01, P < 0.05
Han et al. (2017)	Brain	Neuroinflammatory system	↓TNF-α, IL-1β, IL-6, P < 0.05
Chai (2017)	Brain	1. Amyloid protein system 2. Cholinergic system 3. Mitochondrial system	1. ↓Aβ_42_, P = 0.01 2. ↑Ach, ChAT, P < 0.05 3. ↑Bcl-2/Bax, P < 0.05
Liu (2018)	Brain	Amyloid protein system	↓C99, P < 0.05
Guo et al. (2019)	Brain	1. Tau pathological system 2. Cholinergic system 3. Neuroinflammatory system 4. Mitochondrial system	1. ↓Tau, P < 0.05 2. ↓AChE, P < 0.05 3. ↓TNF-α, IL-1β, P < 0.05 4. ↑Bcl-2/Bax, P < 0.05
Du (2019)	Brain	Neuroinflammatory system	↓TNF-α, IL-1β, IL-6, P < 0.01
Li et al. (2020)	Brain	PI3K/Akt signaling pathway	↑PI3K, p-Akt, P < 0.01
Jiao et al. (2022)	Brain	1. Tau pathological system 2. PI3K/Akt signaling pathway 3. Neuroinflammatory system	1. ↓Tau, p-Tau, P < 0.01 2. ↑p-Akt, ↓p-GSK-3β, P < 0.01, P < 0.05 3. ↓TNF-α, IL-1β, IL-6, NLRP3, P < 0.01, P < 0.05
Wang et al. (2021)	Serum, brain	1. Amyloid protein system 2. Neuroinflammatory system 3. Cholinergic system	1. ↓Aβ_1-40_, P < 0.05 2. ↓TNF-α, IL-1β, IL-6, P < 0.01, P < 0.05 3. ↑Ach, P < 0.01
Liu et al. (2021)	Serum	Oxidative stress system	↓SOD, MPO, iNOS, P < 0.01, P < 0.05
Lou et al. (2022)	Brain	Neuroinflammatory system	↓TNF-α, IL-1β, IL-6, P < 0.01
Liang (2022)	Brain	1. Amyloid protein system 2. Neuroinflammatory system	1. ↓Aβ, P < 0.01 2. ↓Iba1, IL-6, P < 0.05
Pei et al. (2022)	Brain	1. Cholinergic system 2. Neuroinflammatory system 3. Amyloid protein system 4. Tau pathological system 5. Mitochondrial system	1. ↓AchE, ↑Ach, ChAT, P < 0.01, P < 0.05 2. ↓TNF-α, IL-1β, IL-6, P < 0.01, P < 0.05 3. ↓Aβ_1-40_, Aβ_1-42_, P < 0.01, P < 0.05 4. ↓Tau, P < 0.05 5. ↑Bcl-2/Bax, P < 0.05
Shan et al. (2024a)	Serum, brain	1. Oxidative stress system 2. Neuroinflammatory system 3. Cholinergic system 4. Mitochondrial system	1. ↑SOD, ↓MDA, P < 0.01, P < 0.05 2. ↓TNF-α, IL-1β, P < 0.0001 3. ↑Ach, P < 0.01, P < 0.05 4. ↓Bax, P < 0.05
Su et al. (2023)	Brain	1. Oxidative stress system 2. Neurotrophic factor system 3. Neuroinflammatory system	1. ↑SOD, ↓MDA, P < 0.01 2. ↑BDNF, P < 0.05 3. ↓NLRP3, P < 0.05
Li M. (2023)	Serum	1. Neurotrophic factor system 2. Neuroinflammatory system 3. Oxidative stress system	1. ↑BDNF, P < 0.001 2. ↓NLRP3, IL-1β, IL-18, P < 0.05 3. ↑SOD, ↓MDA, P < 0.01, P < 0.00
Lu (2023)	Serum	1. Neuroinflammatory system 2. PI3K/Akt signaling pathway	1. ↓TNF-α, IL-1β, IL-6, P < 0.01, P < 0.05 2. ↓p-GSK-3β, P < 0.01, P < 0.05
Li R. (2023)	Brain	1. Neurotrophic factor system 2. PI3K/Akt signaling pathway 3. Mitochondrial system	1. ↑BDNF, P < 0.05 2. ↑PI3K, ↓p-GSK-3β, P < 0.05 3. ↑Bcl-2/Bax, P < 0.05
Xu et al. (2023)	Serum, brain	1. Oxidative stress system 2. Amyloid protein system 3. Neuroinflammatory system	1. ↑SOD, ↓MDA, P < 0.01, P < 0.05 2. ↓Aβ_40_, Aβ_42_, P < 0.01 3. ↓TNF-α, IL-1β, IL-6, P < 0.01, P < 0.05
Li S. (2023)	Brain	Neuroinflammatory system	↓NLRP3, TNF-α, IL-1β, P < 0.01, P < 0.05
Yan et al. (2024)	Brain	Oxidative stress system	↓MDA, P < 0.01
Wang (2024)	Brain	Neuroinflammatory system	↓TNF-α, IL-1β, IL-6, P < 0.05
Cheng et al. (2024)	Serum	1. Amyloid protein system 2. Neuroinflammatory system	1. ↓Aβ_1-42_, P < 0.01, P < 0.05 2. ↓NLRP3, IL-18, IL-1β, P < 0.01, P < 0.05
Liu (2024)	Brain	Cholinergic system	↓AchE, ↑Ach, P < 0.01
Li et al. (2025)	Brain	1. Oxidative stress system 2. Neuroinflammatory system 3. Cholinergic system 4. Amyloid protein system	1. ↑SOD, ↓MDA, P < 0.0001 2. ↓TNF-α, IL-1β, IL-6, P < 0.0001 3. ↓AchE, P < 0.0001 4. ↓Aβ
Zhou et al. (2025)	Serum	Neuroinflammatory system	↓TNF-α, IL-1β, IL-6, P < 0.0001
Cai et al. (2025)	Serum, brain	Neuroinflammatory system	↓TNF-α, IL-1β, IL-6, P < 0.001, P < 0.0001
Kang et al. (2025)	Brain	Oxidative stress system	↑SOD, P < 0.01, P < 0.05
Yang et al. (2025)	Brain	Neuroinflammatory system	↓TNF-α, IL-1β, IL-6, IL-17, P < 0.01, P < 0.05
Ma et al. (2025)	Serum, brain	1. Neuroinflammatory system 2. Amyloid protein system	1. ↓TNF-α, IL-1β, IL-6, P < 0.01 2. ↓Aβ
Shan et al. (2024b)	Brain	1. Oxidative stress system 2. Neuroinflammatory system 3. Cholinergic system	1. ↑SOD, ↓MDA, P < 0.001 2. ↓TNF-α, IL-1β, IL-6, IL-18, P < 0.001 3. ↓AchE, P < 0.001

Aβ, β-amyloid peptide; β-APP, β-Amyloid Precursor Protein; p-Tau, phosphorylated Tau; 5-HT, 5-hydroxytryptamine; 5-HIAA, 5-Hydroxyindoleacetic Acid; DA, dopamine; NE, norepinephrine; TNF-α, tumor necrosis factor-α; IL-1β, interleukin-1β; IL-6, interleukin-6; IL-8, interleukin-8; IL-18, interleukin-18; IL-17, interleukin-17; NLRP3, NOD-like receptor thermal protein domain-associated protein 3; BDNF, brain-derived neurotrophic factor; MDA, malondialdehyde; SOD, superoxide dismutase; MPO, myeloperoxidase; iNOS, inducible nitric oxide synthase; AchE, acetyl cholinesterase; Ach, acetylcholine; ChAT, choline acetyl cholinesterase; ChE, cholinesterase; Bax, bcl-2-associated X protein; Bcl-2, B-cell lymphoma 2 protein; Caspase-3, cysteine-aspartic acid protease 3; PI3K, phosphatidylinositol 3-kinase; p-Akt, phosphorylated protein kinase B; p-GSK-3β, phosphorylated Glycogen Synthase Kinase-3β; C99, carboxyl-terminal fragment of 99 amino acids.

The pathogenesis of AD mainly involves Aβ, tau, as well as glia (Guo et al., 2020). Notably, several studies have demonstrated that KXS significantly reduces Aβ levels (Shi et al., 2013; Yu, 2015; Li et al., 2016; Wang, 2016; Chai, 2017; Wang et al., 2021; Liang, 2022; Pei et al., 2022; Li et al., 2025; Ma et al., 2025) in brain and modulates tau pathology (Li et al., 2016; Wang, 2016; Guo et al., 2019; Jiao et al., 2022; Pei et al., 2022). Aβ deposition initiates a spectrum of microglia-mediated neuroinflammatory responses. KXS has been shown to significantly reduce the expression of key pro-inflammatory cytokines, mainly including TNF-α, IL-1β, and IL-6 (Shi et al., 2013; Han et al., 2017; Du, 2019; Guo et al., 2019; Wang et al., 2021; Jiao et al., 2022; Liang, 2022; Lou et al., 2022; Pei et al., 2022; Li M., 2023; Li S., 2023; Lu, 2023; Su et al., 2023; Xu et al., 2023; Chen S. et al., 2024; Shan et al., 2024a; Shan et al., 2024b; Wang, 2024; Cai et al., 2025; Li et al., 2025; Ma et al., 2025; Yang et al., 2025; Zhou et al., 2025). Beyond these, a meta-analysis has shown that neuroinflammation, oxidative stress, and mitochondrial dysfunction, play a central role in AD pathogenesis (Song et al., 2021). Oxidative stress plays a critical role in AD and studies have demonstrated that KXS ameliorates oxidative stress by increasing the activity of SOD and decreasing the lipid peroxidation product MDA (Huang et al., 1999; Zhou et al., 2008; Gao et al., 2010; Lv, 2015; Liu et al., 2021; Li S., 2023; Su et al., 2023; Xu et al., 2023; Shan et al., 2024a; Shan et al., 2024b; Yan et al., 2024; Kang et al., 2025; Li et al., 2025). Furthermore, dysfunction of the cholinergic system is also a key pathological feature underlying cognitive decline in AD (Hampel et al., 2018). Additionally, the evidence supports that KXS directly modulates the cholinergic system, mainly increasing ACh levels and decreasing the activity of AchE (Zhong, 2005; Gao et al., 2010; Lv, 2015; Li et al., 2016; Chai, 2017; Guo et al., 2019; Wang et al., 2021; Pei et al., 2022; Liu, 2024; Shan et al., 2024a; Shan et al., 2024b; Li et al., 2025).

## 4 Discussion

### 4.1 Principal findings

This meta-analysis provides a systematic evaluation of the therapeutic effects and potential mechanisms of KXS in preclinical AD models, based on 44 studies involving 2,681 animals. The results demonstrated that KXS significantly improved cognitive function compared to the model group. These cognitive benefits were consistently observed across multiple behavioral tests. The MWM is a well-established experiment for assessing spatial learning and memory (Vorhees and Williams, 2006). Additionally, the NOR test assesses learning and memory in mice through the relative identification index (Leger et al., 2013). Similarly, the Y-maze test evaluates cognitive functions and behavioral patterns by the spontaneous alternation performance (Kraeuter et al., 2019). Treatment with KXS significantly enhanced cognitive performance across a series of behavioral tests. Similarly, the biomarker data revealed that modulated the Aβ and tau protein systems by reducing the levels of Aβ and tau proteins; anti-inflammatory effects by reducing the levels of IL-6, IL-8, IL-1β, and TNF-α; exhibited potent antioxidant properties by decreasing MDA levels and increasing SOD activity; showed effects on the cholinergic system by decreasing AchE levels and increasing ACh content. Due to significant heterogeneity, a random-effects model was applied for data analysis. Subgroup meta-analyses and meta-regression were performed to assess the influence of specific variables and to explore sources of heterogeneity. The SYRCLE assessment indicated unclear risk of bias across most domains, particularly in randomization, allocation concealment, and blinding. Consequently, these findings must be interpreted with extreme caution. This study conducted a systematic review and meta-analysis of the available evidence. However, the certainty of the supporting evidence was low or very low on the basis of GRADE criteria, primarily due to the risk of bias in the design of the included original studies. Consequently, the findings of this meta-analysis should be interpreted with extreme caution.

### 4.2 Potential mechanisms

This meta-analysis showed that KXS significantly reduced the levels of key inflammatory factors, including IL-6, IL-1β, and TNF-α. This finding is critical because neuroinflammation is a key driver of AD pathogenesis (Calsolaro and Edison, 2016). Notably, activated microglia and astrocytes impair Aβ plaques clearance and trigger the release of range of pro-inflammatory cytokines (Singh, 2022; Thakur et al., 2023). A body of evidence suggests the pro-inflammatory cytokines such as TNF-α and IL-6 have been shown to upregulate beta-secretase1 activity and Nuclear Factor Kappa B (NF-κB) expression, thereby promoting the generation of Aβ (Dhapola et al., 2021). Ginsenoside Rb1 from ginseng improves cognitive function, an effect associated with reduced levels of IL-1β and Aβ, suggesting its neuroprotective effects are mediated through anti-inflammatory mechanisms (Lin et al., 2019). Similarly, polygala acid and polysaccharide attenuate neuroinflammatory responses in microglia by reducing TNF-α, and IL-1β (Zeng et al., 2022; Zhao and Jia, 2024). Therefore, the findings suggest that the KXS may alleviates AD pathology by suppressing pro-inflammatory cytokines.

In addition, the meta-analysis indicated that the amelioration of oxidative stress in AD models by KXS was associated with the marked modulation of critical redox markers, evidenced by decreased MDA levels and enhanced SOD activity. Oxidative stress plays a role in AD through multiple pathological mechanisms and directly contributing to neuronal damage (Bai et al., 2022). Crucially, mitochondrial dysfunction and oxidative stress increase both Aβ production and tau phosphorylation, while Aβ and tau pathologies exacerbate redox imbalance (Liu et al., 2023). Therefore, enhancing intrinsic antioxidant defenses, such as SOD, which is crucial for detoxification and redox balance, emerges as a viable treatment avenue (Chidambaram et al., 2024). Poria cocos polysaccharide, has been shown to mitigate oxidative stress by enhancing hippocampal SOD activity and reducing MDA levels (Zhou et al., 2021). Similarly, other key metabolites, including polygala glycoprotein and ginsenoside Rg1, have also been reported to alleviate oxidative stress in AD models (Wu et al., 2022; Cheng et al., 2025). In summary, the findings indicate that KXS alleviates cognitive and pathological deficits in AD by modulating oxidative stress.

Meanwhile, this meta-analysis showed that KXS could decrease AchE content and increase ACh levels. The cholinergic system of the brain plays a pivotal role in cognitive processes, notably in neurodegenerative conditions like AD (Hampel et al., 2018). Thus, enhancing cholinergic neurotransmission by promoting ACh synthesis remains a viable therapeutic strategy (Ferreira-Vieira et al., 2016). Evidence indicates that KXS improves cognition by modulating the cholinergic system (Liu et al., 2025). Beyond the cholinergic system, AD pathology involves complex interactions with adrenergic and glutamatergic systems (Cheng et al., 2021). Studies have established that β2-adrenergic receptor activation increases Aβ production by enhancing gamma-secretase activity (Ni et al., 2006), and subsequently mediates Aβ-induced tau pathology (Wang et al., 2013). Furthermore, Aβ itself can trigger the degradation of β2-adrenergic receptors, thereby compromising both adrenergic and glutamatergic neurotransmission (Wang et al., 2011). Crucially, studies demonstrated that norepinephrine levels in the brain were increased after KXS treatment compared with the model group (Shi et al., 2017b), suggesting a broader modulatory role on neurotransmitter systems. Therefore, the therapeutic effect of KXS extends beyond cholinergic system enhancement to include a multi-system regulatory influence on key pathways implicated in AD.

### 4.3 Challenges and perspectives

The field of TCM pharmacokinetics, rooted in kinetic principles, examines the absorption, distribution, metabolism, and excretion of active metabolites and their associated concentration-efficacy relationships (Li et al., 2015). For instance, it has been demonstrated that ginsenoside Rg1 can enter the hippocampus of AD model rats and significantly promote the release of ACh from this brain region (Liu et al., 2019). Similarly, pharmacokinetic studies have shown that the active metabolites of *A. tatarinowii* Rhizoma, such as asarone, are slowly absorbed after oral administration, while toxicity studies have shown that this botanical drug has no carcinogenic, teratogenic or mutagenic effects (Wen et al., 2023). Furthermore, research indicates that formula compatibility improves the bioavailability of *Polygala tenuifolia* Willd, and alters its pharmacokinetic properties, thereby promoting its pharmacodynamic efficacy (Ba et al., 2012). These findings emphasize the importance of defining pharmacokinetics of KXS for predicting its efficacy and safety.

### 4.4 Strengths and limitations

This is the first comprehensive systematic review and meta-analysis to evaluate the effects of KXS on behavioral and molecular outcomes in animal models of AD. This study ensured comprehensiveness in literature retrieval, screening, data extraction, and bias risk assessment. In addition, to minimize heterogeneity, data for each outcome were rigorously screened prior to pooling in the meta-analysis. However, several limitations should be acknowledged. First, the overall quality of the included original studies was generally low. As indicated by the risk of bias assessment, the majority of studies inadequately described the allocation concealment process, and only a few provided detailed baseline data to demonstrate comparability between groups. According to the GRADE framework, these methodological shortcomings correspond to a high risk of bias, which substantially weakens the credibility of the overall body of evidence. Finally, high heterogeneity was observed in the results, and although meta-subgroup analyses and meta-regressions were performed, the sources of high heterogeneity were still not fully addressed.

## 5 Conclusion

This meta-analysis provides preliminary evidence that KXS may exerts therapeutic effects on AD via multifaceted mechanisms, primarily involving anti-inflammatory and antioxidant activities, modulation of the cholinergic system, and regulation the amyloid and tau protein systems. However, these findings should be interpreted with caution due to the significant heterogeneity observed across the included studies and the generally low methodological quality of the original research. Therefore, high-quality preclinical studies with strict randomization, allocation concealment, and blinding are needed for future research.

## Data Availability

The original contributions presented in the study are included in the article/Supplementary Material, further inquiries can be directed to the corresponding author.
