# Supplementary material for: Efficacy and possible mechanism of Kai-xin-san in animal models of Alzheimer’s disease: a systematic review and meta-analysis of preclinical studies
Source: Front Pharmacol. 2026 Apr 23;17:1806181. doi: 10.3389/fphar.2026.1806181 (PMC13149463; doi:10.3389/fphar.2026.1806181)
Supplement: Supplementary file 1 [file Supplementaryfile1.docx]

Supplementary Material

Content

[1 Supplementary Search strategy 1](#_Toc225018694)

[2 Supplementary Table 2](#_Toc225018695)

[2.1 Table S1 Subgroup meta-analysis. 2](#_Toc225018696)

[2.2 Table S2. The meta regression test. 6](#_Toc225018697)

[2.3 Table S3 Subgroup meta-analysis. 9](#_Toc225018699)

[2.4 Table S4 The GRADE certainty level. 11](#_Toc225018700)

[3 Supplementary Figure 14](#_Toc225018701)

[3.1 Figure S1 Sensitivity analyses. 14](#_Toc225018702)

[3.2 Figure S2 Trim-and-fill analysis. 24](#_Toc225018703)

# 1 Supplementary Search strategy

Web of Science

((((TS=(Kaixinsan)) OR TS=(Kai-xin san)) OR TS=(Kai-xin-san)) AND (((TS=(rodent)) OR TS=(rat)) OR TS=(mice))) OR TS=(mouse)))) AND TS=(Alzheimer)

Pubmed

((Kai xin san[Title/Abstract]) OR (Kai-xin san[Title/Abstract]) OR (Kai-xin-san[Title/Abstract])) AND (Alzheimer[Title/Abstract]) AND ((rodent[Title/Abstract]) OR (rat[Title/Abstract]) OR (mice[Title/Abstract]) OR (mouse[Title/Abstract]) OR (rats[Title/Abstract]))

Embase

kaixinsan AND ('Alzheimer'/exp OR Alzheimer) AND ('animal'/exp OR animal)

CNKI

(TKA='痴呆'OR TKA='健忘' OR TKA='阿尔茨海默') AND (TKA='大鼠' OR TKA='啮齿'OR TKA='动物' OR TKA='实验') AND (TKA='开心散')

WangFang

主题 :(痴呆 OR 健忘 OR 阿尔茨海默) AND 主题 :(大鼠 OR 啮齿 OR 动物 OR 实验) AND 主题 :(开心散)

VIP

R=(痴呆 OR 健忘 OR 阿尔茨海默) AND R=(大鼠 OR 啮齿 OR 动物 OR 实验) AND R=(开心散)

SinoMed

"痴呆" [常用字段:智能] OR "健忘" [常用字段:智能] OR "阿尔茨海默" [常用字段:智能] OR [常用字段:智能] "AD" 开心散" [常用字段:智能] AND "大鼠" [常用字段:智能] OR "啮齿" [常用字段:智能] OR "动物" [常用字段:智能] OR "实验"[常用字段:智能]

# 2 Supplementary Table

## 2.1 Table S1 Subgroup meta-analysis.

| **Table S1 Subgroup meta-analysis.** | | | | | | |
| --- | --- | --- | --- | --- | --- | --- |
| Parameter | Subgroup |  | No. studies | SMD [95% CI] | *I^2^* | *P* |
| Escape latency from the target quadrant | Animal species | Rat | 13 | -1.68 [-2.18, -1.18] | 54.2% | =0.010 |
|  |  | Mouse | 23 | -2.24 [-2.79, -1.70] | 76.7% | <0.001 |
|  | Animal strains | Wistar | 4 | -1.85 [-2.81, -0.89] | 66.3% | =0.031 |
|  |  | KM | 5 | -3.2 [-4.75, -1.66] | 85.8% | <0.001 |
|  |  | SD | 9 | -1.61 [-2.22, -1.00] | 76.7% | =0.033 |
|  |  | APP/PS1 | 13 | -2.30 [-3.13, -1.47] | 78.7% | <0.001 |
|  |  | 5×FAD | 2 | -1.29 [-1.93, -0.66] | 0.0% | =0.923 |
|  |  | ICR | 1 | -1.94 [-3.19, -0.69] | 0.0% | <0.001 |
|  |  | SAMP8 | 2 | -1.56 [-2.33, -0.78] | 0.0% | =0.608 |
|  | Modeling  method | Chemical-induced | 19 | -2.08 [-2.60, -1.55] | 71.4% | <0.001 |
|  |  | Transgenic | 15 | -2.06 [-2.73, -1.39] | 75.3% | <0.001 |
|  |  | SAMP8 | 2 | -1.56 [-2.33, -0.78] | 0.0% | =0.608 |
| Entry frequency into the target quadrant | Animal species | Rat | 13 | 1.27 [0.97, 1.58] | 1.4% | =0.432 |
|  |  | Mouse | 22 | 2.06 [1.51, 2.60] | 76.2% | <0.001 |
|  | Animal strains | Wistar | 3 | 1.29 [0.73, 1.84] | 0.0% | =0.611 |
|  |  | KM | 3 | 3.35 [1.74, 4.05] | 71.5% | =0.030 |
|  |  | SD | 10 | 1.30 [0.89, 1.71] | 19.5% | =0.264 |
|  |  | APP/PS1 | 12 | 2.26 [1.41, 3.11] | 78.0% | <0.001 |
|  |  | 5×FAD | 2 | 1.05 [0.13, 1.06] | 54.2% | =0.139 |
|  |  | ICR | 2 | 2.75 [1.47, 6.97] | 91.9% | <0.001 |
|  |  | SAMP8 | 3 | 1.20 [0.62, 1.78] | 0.0% | =0.912 |
|  | Modeling  method | Chemical-induced | 18 | 1.70 [1.24, 2.16] | 63.7% | <0.001 |
|  |  | Transgenic | 12 | 1.79 [1.11, 2.47] | 72.5% | <0.001 |
|  |  | SAMP8 | 5 | 1.73 [0.67, 2.79] | 73.3% | <0.001 |
| The crossing distance in the target quadrant | Animal species | Rat | 10 | 0.58 [-0.55, 1.70] | 84.8% | =0.932 |
|  |  | Mouse | 7 | 0.65 [-0.73, 2.04] | 91.8% | <0.001 |
|  | Animal strains | KM | 3 | 0.02 [-2.73, 2.76] | 94.9% | <0.001 |
|  |  | SD | 6 | 0.58 [-0.55, 1.70] | 84.8% | <0.001 |
|  |  | APP/PS1 | 5 | 1.85 [-0.87, 4.58] | 92.8% | <0.001 |
|  |  | ICR | 1 | -1.81 [-3.03, -0.60] | 0.0% | <0.001 |
|  |  | SAMP8 | 1 | 0.80 [-0.12, 1.72] | 0.0% | <0.001 |
|  | Modeling method | Chemical-induced | 11 | 0.22 [-0.83, 1.26] | 89.4% | <0.001 |
|  |  | Transgenic | 5 | 1.85 [-0.87, 4.58] | 92.8% | <0.001 |
|  |  | SAMP8 | 1 | 0.80 [-0.12, 1.72] | 0.0% | <0.001 |
| Time spent in the target quadrant | Animal species | Rat | 8 | 1.28 [0.68, 1.87] | 52.1% | =0.932 |
|  |  | Mouse | 18 | 2.71 [1.40, 2.94] | 85.4% | <0.001 |
|  | Animal strains | Wistar | 1 | 2.10 [1.07, 3.13] | 0.0% | <0.001 |
|  |  | KM | 3 | 4.90 [1.45, 8.35] | 92.6% | <0.001 |
|  |  | SD | 7 | 1.13 [0.52, 1.74] | 46.7% | =0.081 |
|  |  | APP/PS1 | 11 | 2.39 [1.22, 3.57] | 87.1% | <0.001 |
|  |  | 5×FAD | 2 | 0.74 [0.15, 1.33] | 0.0% | =0.360 |
|  |  | SAMP8 | 2 | 1.67 [0.73, 2.61] | 42.9% | =0.186 |
|  | Modeling  method | Chemical-induced | 11 | 2.71 [1.40, 2.94] | 78.4% | <0.001 |
|  |  | Transgenic | 13 | 2.71 [1.40, 2.94] | 85.3% | <0.001 |
|  |  | SAMP8 | 2 | 2.71 [1.40, 2.94] | 42.9% | =0.186 |
| NOR | Animal species | Rat | 1 | 1.84 [0.52, 3.16] | 0.0% | <0.001 |
|  |  | Mouse | 9 | 1.79 [1.15, 2.44] | 65.0% | =0.004 |
|  | Animal strains | SD | 1 | 1.84 [0.52, 3.16] | 0.0% | <0.001 |
|  |  | APP/PS1 | 5 | 2.75 [1.62, 3.89] | 65.4% | =0.021 |
|  |  | 5×FAD | 3 | 1.02 [0.51, 1.53] | 0.0% | =0.800 |
|  |  | SAMP8 | 1 | 1.14 [0.27, 2.02] | 0.0% | <0.001 |
|  | Modeling  method | Chemical-induced | 1 | 1.84 [0.52, 3.16] | 0.0% | <0.001 |
|  |  | Transgenic | 8 | 1.93 [1.19, 2.68] | 68.4% | =0.002 |
|  |  | SAMP8 | 1 | 1.14 [0.27, 2.02] | 0.0% | <0.001 |
| Y-maze | Animal species | Rat | 1 | 3.18 [1.44, 4.92] | 0.0% | <0.001 |
|  |  | Mouse | 7 | 1.98 [1.01, 2.96] | 77.3% | <0.001 |
|  | Animal strains | SD | 1 | 3.18 [1.44, 4.92] | 0.0% | <0.001 |
|  |  | APP/PS1 | 5 | 2.89 [1.29, 4.48] | 83.4% | <0.001 |
|  |  | 5×FAD | 2 | 1.05 [0.41, 1.69] | 0.0% | =0.724 |
|  | Modeling  method | Chemical-induced | 1 | 3.18 [1.44, 4.92] | 0.0% | <0.001 |
|  |  | Transgenic | 7 | 1.98 [1.01, 2.96] | 77.3% | <0.001 |
| Aβ in the hippocampus | Animal species | Rat | 2 | -1.55 [-2.56, -0.53] | 0.0% | =0.939 |
|  |  | Mouse | 7 | -2.66 [-3.76, -1.56] | 61.3% | =0.017 |
|  | Animal strains | Wistar | 1 | -1.50 [-3.00, -0.00] | 0.0% | <0.001 |
|  |  | KM | 1 | -3.82 [-5.41, -2.24] | 0.0% | <0.001 |
|  |  | SD | 1 | -1.58 [-2.95, -0.21] | 0.0% | <0.001 |
|  |  | APP/PS1 | 5 | -2.53 [-4.14, -0.93] | 64.9% | =0.022 |
|  |  | 5×FAD | 1 | -2.52 [-3.76, -1.28] | 0.0% | <0.001 |
|  | Modeling  method | Chemical-induced | 3 | -2.27 [-3.70, -0.83] | 64.5% | =0.060 |
|  |  | Transgenic | 6 | -2.39 [-3.57, -1.22] | 57.9% | =0.036 |
| Tau in the hippocampus | Animal species | Rat | 2 | -2.85 [-5.07, -0.63] | 78.9% | =0.009 |
|  |  | Mouse | 3 | -2.51 [-4.74, -0.28] | 62.3% | =0.104 |
|  | Animal strains | KM | 1 | -4.42 [-6.18, -2.66] | 0.0% | <0.001 |
|  |  | SD | 2 | -2.51 [-4.74, -0.28] | 62.3% | =0.104 |
|  |  | APP/PS1 | 1 | -1.03 [-2.40, 0.34] | 0.0% | <0.001 |
|  |  | SAMP8 | 1 | -3.32 [-5.57, -1.08] | 0.0% | <0.001 |
|  | Modeling  method | Chemical-induced | 3 | -3.18 [-5.04, -1.31] | 66.7% | =0.050 |
|  |  | Transgenic | 2 | -2.00 [-4.22, -0.22] | 65.7% | =0.088 |
| TNF-α in the serum | Animal species | Rat | 3 | -2.27 [-3.41, -1.13] | 18.3% | =0.294 |
|  |  | Mouse | 5 | -2.06 [-4.47, 3.07] | 91.5% | <0.001 |
|  | Animal strains | SD | 3 | -2.27 [-3.41, -1.13] | 18.3% | =0.294 |
|  |  | APP/PS1 | 4 | -2.88 [-4.38, -1.38] | 63.5% | =0.042 |
|  |  | SAMP8 | 1 | 2.03 [0.90, 3.15] | 0.0% | <0.001 |
|  | Modeling method | Chemical-induced | 3 | -2.27 [-3.41, -1.13] | 18.3% | =0.294 |
|  |  | Transgenic | 4 | -2.88 [-4.38, -1.38] | 63.5% | =0.042 |
|  |  | SAMP8 | 1 | 2.03 [0.90, 3.15] | 0.0% | <0.001 |
| TNF-α in the hippocampus | Animal species | Rat | 3 | -2.29 [-4.38, -0.19] | 78.0% | =0.011 |
|  |  | Mouse | 8 | -3.21 [-4.69, -1.73] | 80.8% | <0.001 |
|  | Animal strains | SD | 3 | -2.29 [-4.38, -0.19] | 78.0% | =0.011 |
|  |  | APP/PS1 | 5 | -2.84 [-4.65, -1.03] | 79.1% | =0.001 |
|  |  | ICR | 1 | -3.61 [-5.35, -1.87] | 0.0% | <0.001 |
|  |  | SAMP8 | 2 | -4.23 [-10.21, 1.74] | 93.4% | <0.001 |
|  | Modeling method | Chemical-induced | 4 | -2.63 [-4.42, -0.83] | 77.9% | =0.004 |
|  |  | Transgenic | 5 | -2.84 [-4.65, -1.03] | 79.1% | <0.001 |
|  |  | SAMP8 | 2 | -4.23 [-10.21, 1.74] | 93.4% | <0.001 |
| IL-1β in the hippocampus | Animal species | Rat | 3 | -3.95 [-7.83, -0.07] | 88.6% | <0.001 |
|  |  | Mouse | 8 | -3.71 [-5.65, -1.77] | 83.9% | <0.001 |
|  | Animal strains | KM | 1 | -8.51 [-16.87, -0.14] | 0.0% | <0.001 |
|  |  | SD | 3 | -3.95 [-7.83, -0.07] | 88.6% | <0.001 |
|  |  | APP/PS1 | 5 | -3.84 [-6.32, -1.36] | 83.7% | <0.001 |
|  |  | ICR | 1 | -5.80 [-8.32, -3.29] | 0.0% | <0.001 |
|  |  | SAMP8 | 1 | -0.29 [-1.54, 0.96] | 0.0% | <0.001 |
|  | Modeling  method | Chemical-induced | 5 | -4.84 [-8.06, -1.62] | 86.3% | <0.001 |
|  |  | Transgenic | 5 | -3.84 [-6.32, -1.36] | 83.7% | <0.001 |
|  |  | SAMP8 | 1 | -0.29 [-1.54, 0.96] | 0.0% | <0.001 |
| IL-6 in the hippocampus | Animal species | Rat | 1 | -0.78 [-1.97, 0.42] | 0.0% | <0.001 |
|  |  | Mouse | 7 | -3.68 [-5.34, -2.02] | 82.3% | <0.001 |
|  | Animal strains | SD | 1 | -0.78 [-1.97, 0.42] | 0.0% | <0.001 |
|  |  | APP/PS1 | 4 | -4.47 [-7.33, -1.62] | 85.2% | <0.001 |
|  |  | 5×FAD | 1 | -1.49 [-2.50, -0.47] | 0.0% | <0.001 |
|  |  | ICR | 1 | -7.45 [-10.57, -4.32] | 0.0% | <0.001 |
|  |  | SAMP8 | 1 | -1.88 [-3.51, -0.25] | 0.0% | <0.001 |
|  | Modeling  method | Chemical-induced | 2 | -3.95 [-10.48, 2.58] | 93.4% | <0.001 |
|  |  | Transgenic | 5 | -3.46 [-5.35, -1.54] | 82.6% | <0.001 |
|  |  | SAMP8 | 1 | -1.88 [-3.51, -0.25] | 0.0% | <0.001 |
| SOD in the serum | Animal species | Rat | 3 | 5.64 [1.63, 9.65] | 87.0% | <0.001 |
|  |  | Mouse | 1 | 4.31 [2.75, 5.87] | 0.0% | <0.001 |
|  | Animal strains | ICR | 1 | 4.31 [2.75, 5.87] | 0.0% | <0.001 |
|  |  | SD | 3 | 5.64 [1.63, 9.65] | 87.0% | <0.001 |
| SOD in the hippocampus | Animal species | APP/PS1 | 2 | 1.26 [0.47, 2.05] | 0.0% | =0.349 |
|  |  | ICR | 7 | 3.15 [1.89, 4.41] | 77.5% | <0.001 |
|  | Animal strains | KM | 2 | 4.38 [-0.83, 9.59] | 95.2% | <0.001 |
|  |  | SD | 2 | 1.26 [0.47, 2.05] | 0.0% | =0.349 |
|  |  | APP/PS1 | 4 | 2.82 [1.62, 4.02] | 30.4% | =0.230 |
|  |  | ICR | 1 | 1.98 [0.97, 2.99] | 0.0% | <0.001 |
|  | Modeling  method | Chemical-induced | 5 | 2.52 [1.12, 3.93] | 85.7% | <0.001 |
|  |  | Transgenic | 4 | 2.82 [1.62, 4.02] | 30.4% | =0.230 |
| MDA in the hippocampus | Animal species | Rat | 1 | -1.90 [-2.99, -0.80] | 0.0% | <0.001 |
|  |  | Mouse | 7 | -3.89 [-6.09, -1.69] | 91.5% | <0.001 |
|  | Animal strains | KM | 2 | -0.17 [-1.01, 0.66] | 94.5% | <0.001 |
|  |  | SD | 1 | -1.90 [-2.99, -0.80] | 0.0% | <0.001 |
|  |  | APP/PS1 | 4 | -3.84 [-6.56, -1.12] | 82.4% | <0.001 |
|  |  | ICR | 1 | -0.17 [-1.01, 0.66] | 0.0% | <0.001 |
|  | Modeling  method | Chemical-induced | 4 | -3.31 [-5.83, -0.79] | 94.0% | <0.001 |
|  |  | Transgenic | 4 | -3.84 [-6.56, -1.12] | 82.4% | <0.001 |
| ACh in the hippocampus | Animal species | Rat | 2 | 6.98 [-4.73, 18.69] | 91.7% | <0.001 |
|  |  | Mouse | 4 | 0.99 [0.04, 1.94] | 60.9% | =0.053 |
|  | Animal strains | KM | 1 | 0.49 [-0.04, 1.38] | 0.0% | <0.001 |
|  |  | SD | 2 | 6.98 [-4.73, 18.69] | 91.7% | <0.001 |
|  |  | APP/PS1 | 3 | 1.34 [-0.13, 2.82] | 71.9% | =0.029 |
|  | Modeling  method | Chemical-induced | 3 | 2.53 [-0.23, 5.28] | 86.7% | <0.001 |
|  |  | Transgenic | 3 | 1.34 [-0.13, 2.82] | 71.9% | =0.029 |
| AchE in the hippocampus | Animal species | Rat | 3 | -5.87 [-12.55, 0.80] | 93.4% | <0.001 |
|  |  | Mouse | 6 | -4.50 [-7.28, -1.72] | 93.4% | <0.001 |
|  | Animal strains | Wistar | 1 | -10.41 [-14.52, -6.31] | 0.0% | <0.001 |
|  |  | KM | 2 | -5.07 [-11.51, 1.63] | 95.8% | <0.001 |
|  |  | SD | 2 | -3.68 [-10.44, 3.07] | 91.5% | <0.001 |
|  |  | APP/PS1 | 4 | -4.37 [-8.22, -0.51] | 92.1% | <0.001 |
|  | Modeling  method | Chemical-induced | 5 | -5.31 [-8.52, -2.11] | 93.0% | <0.001 |
|  |  | Transgenic | 4 | -4.37 [-8.22, -0.51] | 92.1% | <0.001 |

Notes: NOR, novel object recognition; TNF-α, tumor necrosis factor-α; IL-1β, interleukin-1β; IL-6, interleukin-6; MDA, malondialdehyde; SOD, superoxide dismutase; AchE, acetyl cholinesterase; ACh, acetylcholine; Aβ, β-amyloid peptide.

## 2.2 Table S2. The meta regression test.

| Table S2. The meta regression test. | | | | | | |
| --- | --- | --- | --- | --- | --- | --- |
| Metareg | _ES | No. studies | Coef. | t | *P*> t | [95% Conf. Interval] |
| Escape latency from the target quadrant | Animal species | 36 | -0.4112442 | -0.86 | 0.396 | [-1.383417, 0.5609287] |
|  | Animal strains |  | -0.1055998 | -0.96 | 0.346 | [-.3300652, 0.1188655] |
|  | Modeling  method |  | 0.139779 | 0.36 | 0.721 | [-0.6504032, 0.9299611] |
| Entry frequency into the target quadrant | Animal species | 35 | 0.40377 | 0.86 | 0.395 | [-0.549938, 1.357478] |
|  | Animal strains |  | 0.0674003 | 0.58 | 0.565 | [-0.1682385, 0.3030391] |
|  | Modeling  method |  | -0.0037778 | -0.01 | 0.991 | [-0.6654561, 0.6579005] |
| The crossing distance in the target quadrant | Animal species | 17 | 0.5560385 | 0.93 | 0.365 | [-0.7137757, 1.825853] |
|  | Animal strains |  | -0.1607198 | -0.12 | 0.906 | [-3.016142, 2.694703] |
|  | Modeling  method |  | 0.8616708 | 0.80 | 0.435 | [-1.428738, 3.152079] |
| Time spent in the target quadrant | Animal species | 26 | 0.7860993 | 0.79 | 0.435 | [-1.257839, 2.830038] |
|  | Animal strains |  | 0.252018 | 0.78 | 0.445 | [-0.4175788, 0.9216148] |
|  | Modeling  method |  | -0.0377678 | -0.05 | 0.961 | [-1.597358, 1.521822] |
| NOR | Animal species | 10 | -0.0031842 | -0.00 | 0.998 | [-3.043031, 3.036663] |
|  | Animal strains |  | 0.2380851 | 0.59 | 0.573 | [-0.6971075, 1.1732781] |
|  | Modeling  method |  | -0.2087518 | -0.34 | 0.739 | [-1.604233, 1.186729] |
| Y-maze | Animal species | 8 | 0.5225399 | 0.17 | 0.874 | [-7.217218, 8.262298] |
|  | Animal strains |  | 1.332237 | 0.86 | 0.425 | [-2.478917, 5.143391] |
|  | Modeling  method |  | 0.5225399 | 0.17 | 0.874 | [-7.217218, 8.262298] |
| Aβ in the hippocampus | Animal species | 9 | -0.606866 | -0.33 | 0.749 | [-4.909345, 3.695613] |
|  | Animal strains |  | -2.147623 | -1.73 | 0.128 | [-5.086953, 0.7917061] |
|  | Modeling  method |  | -2.213485 | -1.22 | 0.262 | [-6.501563, 2.074594] |
| Tau in the hippocampus | Animal species | 5 | -2.228576 | -0.81 | 0.477 | [-10.98846, 6.531306] |
|  | Modeling  method |  | -4.352994 | -1.99 | 0.140 | [-11.30707, 2.601085] |
| TNF-α in the serum | Animal species | 8 | -0.4598326 | -0.26 | 0.802 | [-4.760498, 3.840833] |
|  | Animal strains |  | -1.563263 | -1.47 | 0.191 | [-4.161511, 1.034985] |
|  | Modeling  method |  | 1.563263 | 1.47 | 0.191 | [-1.034985, 4.161511] |
| TNF-α in the hippocampus | Animal species | 11 | 0.7478116 | 0.43 | 0.676 | [-3.173402, 4.669026] |
|  | Animal strains |  | 0.5130419 | 0.61 | 0.557 | [-1.391213, 2.417297] |
|  | Modeling  method |  | -0.0285627 | -0.03 | 0.974 | [-1.984997, 1.927871] |
| IL-1β in the hippocampus | Animal species | 11 | -0.0726896 | -0.03 | 0.976 | [-5.383681, 5.238302] |
|  | Animal strains |  | 1.257443 | 1.40 | 0.196 | [-0.7785648, 3.293451] |
|  | Modeling  method |  | 1.75092 | 1.19 | 0.266 | [-1.587708, 5.089547] |
| IL-6 in the hippocampus | Animal species | 8 | 1.118805 | 1.06 | 0.329 | [-1.460723, 3.698334] |
|  | Animal strains |  | 3.267846 | 0.93 | 0.388 | [-5.333341, 11.86903] |
|  | Modeling  method |  | 0.7450398 | 0.35 | 0.739 | [-4.483521, 5.9736] |
| SOD in the serum | Animal species | 4 | -1.50565 | -0.28 | 0.804 | [-24.36665, 21.35535] |
|  | Animal strains |  | -1.50565 | -0.28 | 0.804 | [-24.36665, 21.35535] |
| SOD in the hippocampus | Animal species | 9 | 0.434977 | 0.88 | 0.409 | [-0.7361971, 1.606151] |
|  | Animal strains |  | 1.776335 | 1.25 | 0.251 | [-1.582505, 5.135175] |
|  | Modeling  method |  | 0.4867029 | 0.35 | 0.738 | [-2.816037, 3.789443] |
| MDA in the hippocampus | Animal species | 8 | -2.072866 | -0.58 | 0.585 | [-10.87163, 6.725903] |
|  | Animal strains |  | -1.016507 | -0.80 | 0.452 | [-4.108185, 2.07517] |
|  | Modeling  method |  | -0.5569352 | -0.22 | 0.832 | [-6.715824, 5.601954] |
| ACh in the hippocampus | Animal species | 6 | 3.998712 | 1.14 | 0.317 | [-5.712724, 13.71015] |
|  | Animal strains |  | 2.509974 | 1.07 | 0.343 | [-3.977644, 8.997592] |
|  | Modeling  method |  | -2.079792 | -0.57 | 0.600 | [-12.24615, 8.086566] |
| AchE in the hippocampus | Animal species | 9 | 0.9931837 | 0.30 | 0.772 | [-6.797934, 8.784302] |
|  | Animal strains |  | 1.709896 | 1.08 | 0.317 | [-2.044029, 5.463821] |
|  | Modeling  method |  | 0.8851064 | 0.28 | 0.785 | [-6.481376, 8.251589] |

Notes: NOR, novel object recognition; TNF-α, tumor necrosis factor-α; IL-1β, interleukin-1β; IL-6, interleukin-6; MDA, malondialdehyde; SOD, superoxide dismutase; AchE, acetyl cholinesterase; ACh, acetylcholine; Aβ, β-amyloid peptide.

## 2.3 Table S3 Subgroup meta-analysis.

| **Table S3 Subgroup meta-analysis.** | | | | | | |
| --- | --- | --- | --- | --- | --- | --- |
| Parameter | Subgroup |  | No. studies | SMD [95% CI] | *I^2^* | *P* |
| Escape latency from the target quadrant | Baseline characteristics | Low risk | 5 | -0.97 [-1.39, -0.56] | 0.0% | =0.607 |
|  |  | Unclear risk | 31 | -2.25 [-2.70, -1.81] | 71.9% | <0.001 |
| Entry frequency into the target quadrant | Baseline characteristics | Low risk | 5 | 1.17 [0.62, 1.67] | 25.6% | =0.251 |
|  |  | Unclear risk | 30 | 1.85 [1.44, 2.26] | 69.4% | <0.001 |
| The crossing distance in the target quadrant | Baseline characteristics | Low risk | 2 | 1.14 [0.46, 1.82] | 0.0% | =0.638 |
|  |  | Unclear risk | 15 | 0.52 [-0.53, 1.57] | 90.5% | <0.001 |
| Time spent in the target quadrant | Baseline characteristics | Low risk | 3 | 1.19 [0.65, 1.75] | 0.0% | =0.900 |
|  |  | Unclear risk | 23 | 2.00 [1.36, 2.65] | 83.2% | <0.001 |
| NOR | Baseline characteristics | Low risk | 1 | 0.85 [0.01, 1.69] | 0.0% | <0.001 |
|  |  | Unclear risk | 9 | 1.92 [1.28, 2.56] | 60.6% | =0.009 |
| Y-maze | Baseline characteristics | Low risk | 1 | 0.95 [0.10, 1.80] | 0.0% | <0.001 |
|  |  | Unclear risk | 7 | 2.46 [1.34, 3.58] | 78.5% | <0.001 |
| Aβ in the hippocampus | Baseline characteristics | Low risk | 4 | -3.15 [-5.19, -1.11] | 79.3% | =0.002 |
|  |  | Unclear risk | 5 | -2.01 [-2.68, -1.34] | 0.0% | =0.650 |
| Tau in the hippocampus | Baseline characteristics | Low risk | 2 | -2.93 [-5.83, -0.04] | 82.0% | =0.018 |
|  |  | Unclear risk | 3 | -2.53 [-4.39, -0.67] | 65.1% | =0.057 |
| TNF-α in the hippocampus | Baseline characteristics | Low risk | 1 | -1.06 [-2.30, 0.18] | 0.0% | <0.001 |
|  |  | Unclear risk | 10 | -3.21 [-4.51, -1.90] | 80.1% | <0.001 |
| IL-1β in the hippocampus | Baseline characteristics | Low risk | 1 | -0.53 [-1.69, 0.63] | 0.0% | <0.001 |
|  |  | Unclear risk | 10 | -4.12 [-5.92, -2.33] | 83.6% | <0.001 |
| IL-6 in the hippocampus | Baseline characteristics | Low risk | 1 | -0.78 [-1.97, 0.42] | 0.0% | <0.001 |
|  |  | Unclear risk | 7 | -3.68 [-5.34, -2.02] | 82.3% | <0.001 |
| SOD in the serum | Baseline characteristics | Low risk | 1 | 2.69 [0.96, 4.43] | 0.0% | <0.001 |
|  |  | Unclear risk | 3 | 6.08 [2.71, 9.45] | 82.7% | =0.003 |
| ACh in the hippocampus | Baseline characteristics | Low risk | 2 | 0.84 [-0.06, 1.74] | 26.7% | =0.243 |
|  |  | Unclear risk | 4 | 2.56 [0.26, 4.86] | 85.4% | <0.001 |
| AchE in the hippocampus | Baseline characteristics | Low risk | 2 | -1.19 [-2.57, 0.19] | 67.0% | =0.082 |
|  |  | Unclear risk | 7 | -6.37 [-10.06, -2.68] | 94.1% | <0.001 |

Notes: NOR, novel object recognition; TNF-α, tumor necrosis factor-α; IL-1β, interleukin-1β; IL-6, interleukin-6; SOD, superoxide dismutase; AchE, acetyl cholinesterase; ACh, acetylcholine; Aβ, β-amyloid peptide.

## 2.4 Table S4 The GRADE certainty level.

| **Table S4. The GRADE certainty level.** | | | | | | |
| --- | --- | --- | --- | --- | --- | --- |
| Certainty assessment | | | | | | Certainty |
| Outcome | Risk of bias | Inconsistency | Indirectness | Imprecision | Other considerations |  |
| Escape latency from the target quadrant | serious ^a^ | not serious | not serious | not serious | publication bias strongly suspected | ⨁⨁◯◯ Low |
| Entry frequency into the target quadrant | serious ^a^ | not serious | not serious | not serious | publication bias strongly suspected | ⨁⨁◯◯ Low |
| The crossing distance in the target quadrant | serious ^a^ | serious ^b^ | not serious | serious ^c^ | publication bias strongly suspected | ⨁◯◯◯ Very low |
| Time spent in the target quadrant | serious ^a^ | serious ^b^ | not serious | not serious | publication bias strongly suspected | ⨁◯◯◯ Very low |
| NOR | serious ^a^ | not serious | not serious | not serious | publication bias strongly suspected | ⨁⨁◯◯ Low |
| Y-maze | serious ^a^ | not serious | not serious | not serious | publication bias strongly suspected | ⨁⨁◯◯ Low |
| Aβ in the hippocampus | serious ^a^ | serious ^b^ | not serious | not serious | none | ⨁⨁◯◯ Low |
| Tau in the hippocampus | serious ^a^ | not serious | not serious | not serious | none | ⨁⨁⨁◯ Moderate |
| TNF-α in the serum | serious ^a^ | serious ^b^ | not serious | not serious | publication bias strongly suspected | ⨁◯◯◯ Very low |
| TNF-α in the hippocampus | serious ^a^ | not serious | not serious | not serious | publication bias strongly suspected | ⨁⨁◯◯ Low |
| IL-1β in the serum | serious ^a^ | not serious | not serious | not serious | publication bias strongly suspected | ⨁⨁◯◯ Low |
| IL-1β in the hippocampus | serious ^a^ | not serious | not serious | not serious | publication bias strongly suspected | ⨁⨁◯◯ Low |
| IL-6 in the serum | serious ^a^ | not serious | not serious | not serious | publication bias strongly suspected | ⨁⨁◯◯ Low |
| IL-6 in the hippocampus | serious ^a^ | not serious | not serious | not serious | publication bias strongly suspected | ⨁⨁◯◯ Low |
| SOD in the serum | serious ^a^ | serious ^b^ | not serious | not serious | publication bias strongly suspected | ⨁◯◯◯ Very low |
| SOD in the hippocampus | serious ^a^ | serious ^b^ | not serious | not serious | publication bias strongly suspected | ⨁◯◯◯ Very low |
| MDA in the serum | serious ^a^ | not serious | not serious | not serious | publication bias strongly suspected | ⨁⨁◯◯ Low |
| MDA in the hippocampus | serious ^a^ | serious ^b^ | not serious | not serious | publication bias strongly suspected | ⨁◯◯◯ Very low |
| ACh in the hippocampus | serious ^a^ | serious ^b^ | not serious | not serious | publication bias strongly suspected | ⨁◯◯◯ Very low |
| AchE in the hippocampus | serious ^a^ | not serious | not serious | not serious | publication bias strongly suspected | ⨁⨁◯◯ Low |

Notes: ^a^ Most studies presented uncertain risk of bias; ^b^ *I^2^*>50%, There was heterogeneity in the studies; ^c^ The confidence intervals include zero; NOR, novel object recognition; TNF-α, tumor necrosis factor-α; IL-1β, interleukin-1β; IL-6, interleukin-6; MDA, malondialdehyde; SOD, superoxide dismutase; AchE, acetyl cholinesterase; ACh, acetylcholine; Aβ, β-amyloid peptide.

# 3 Supplementary Figure

## 3.1 Figure S1 Sensitivity analyses.


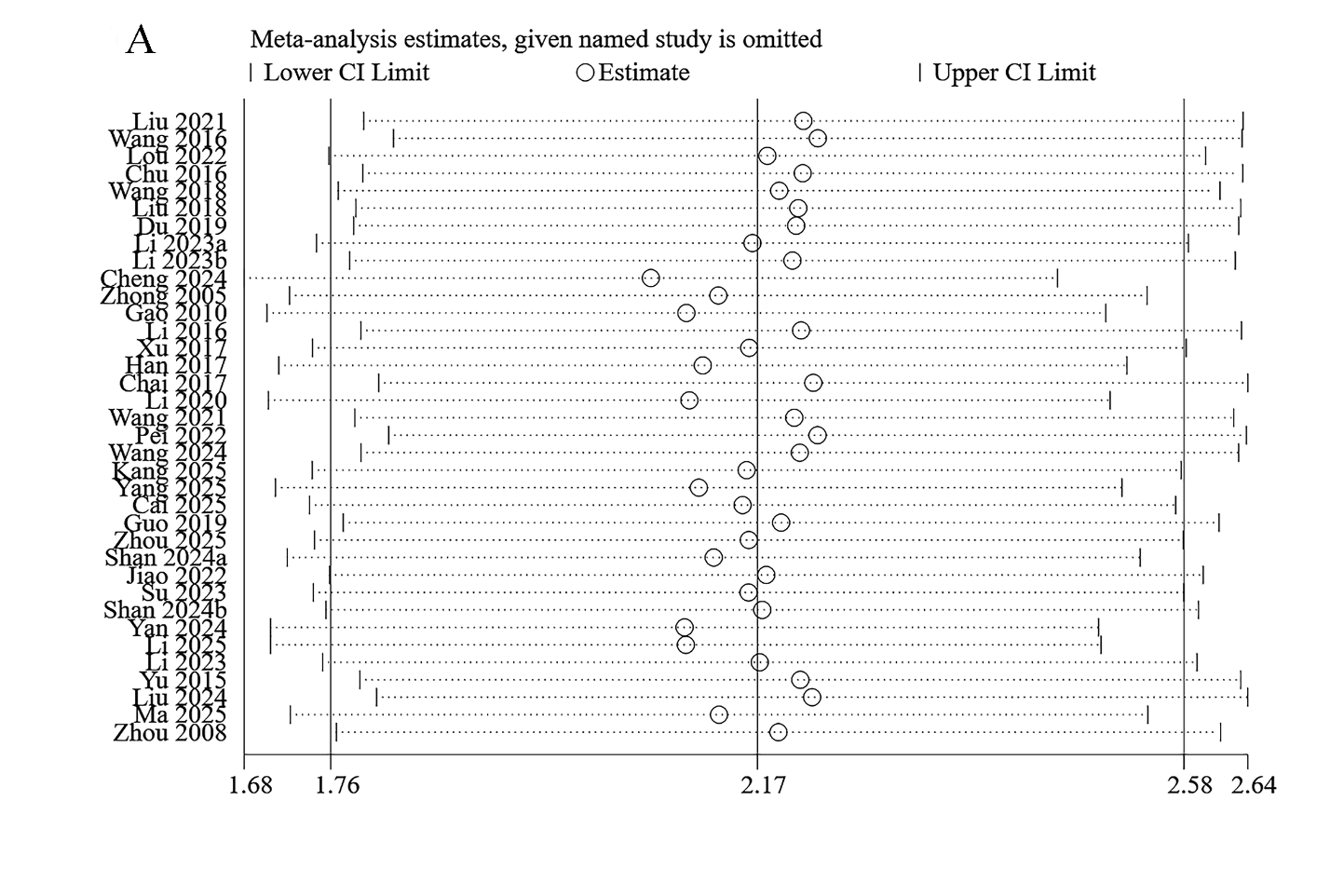


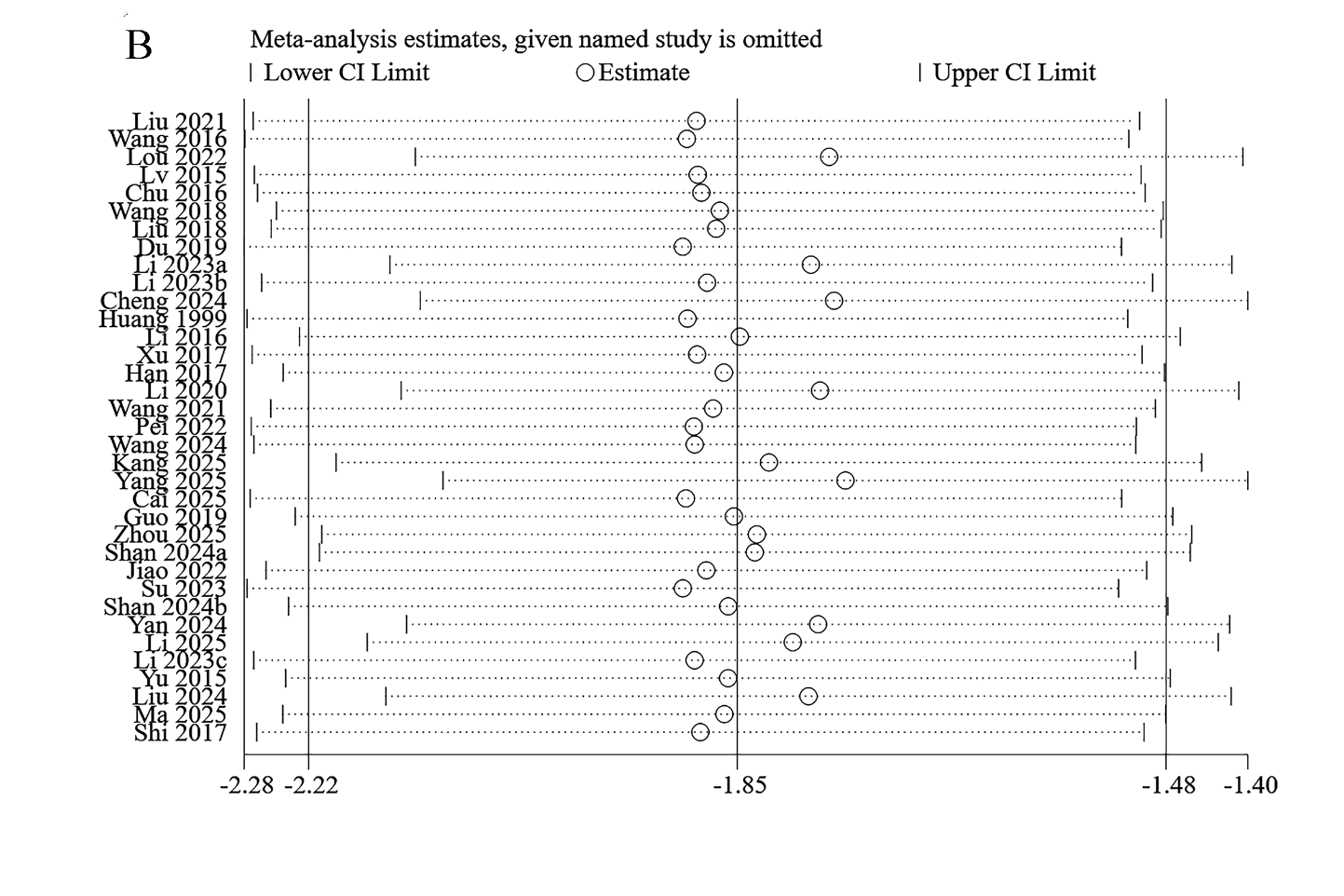


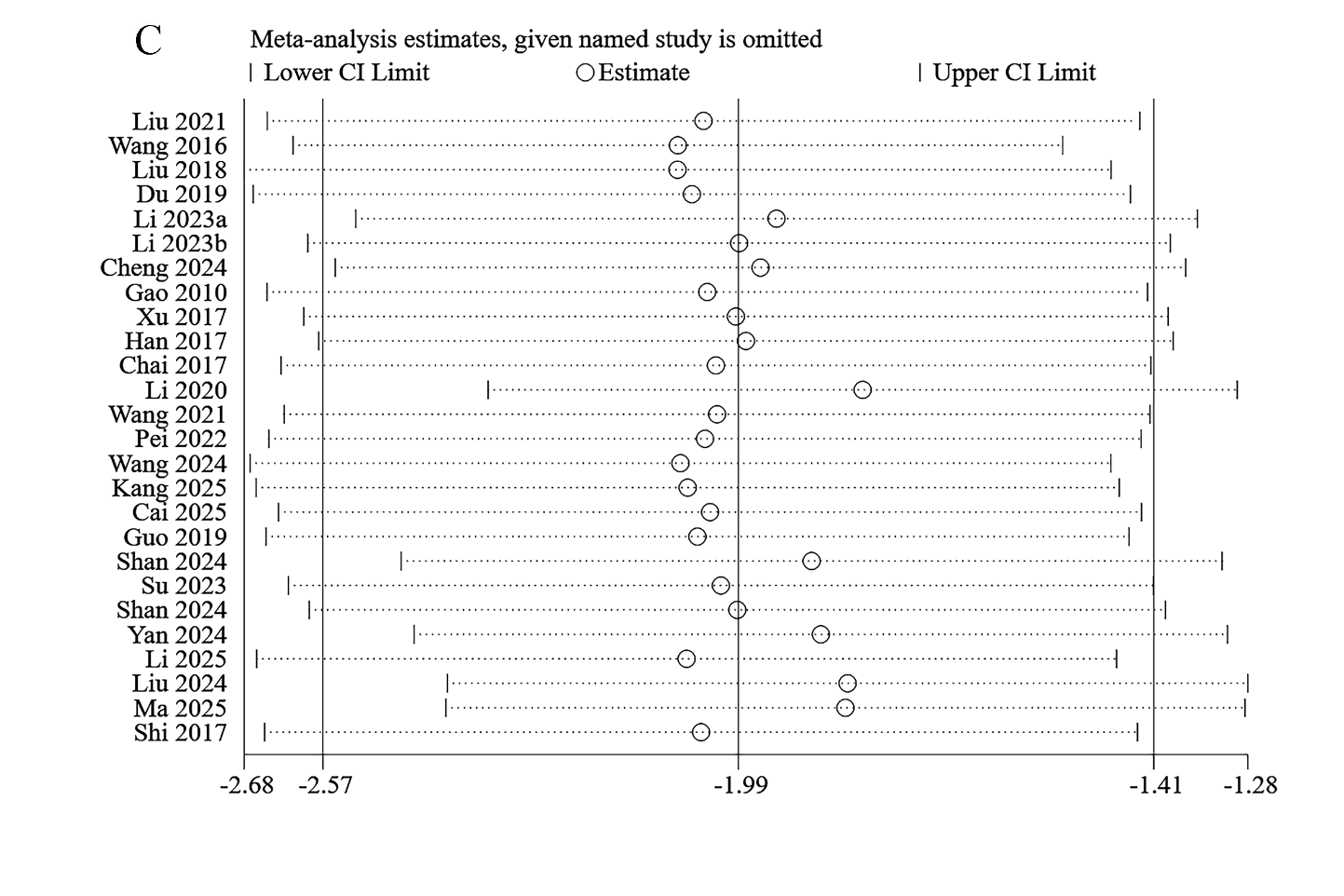


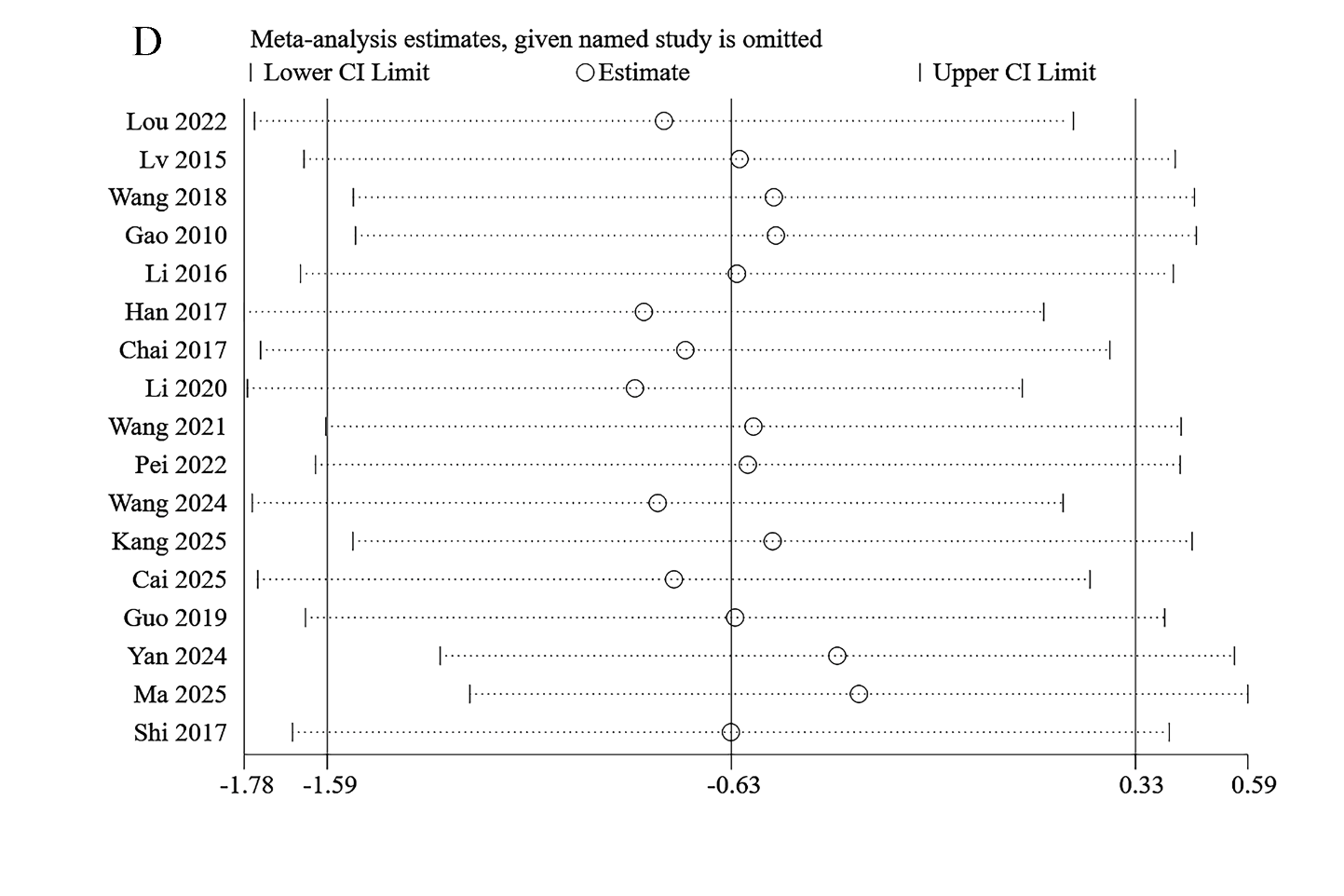


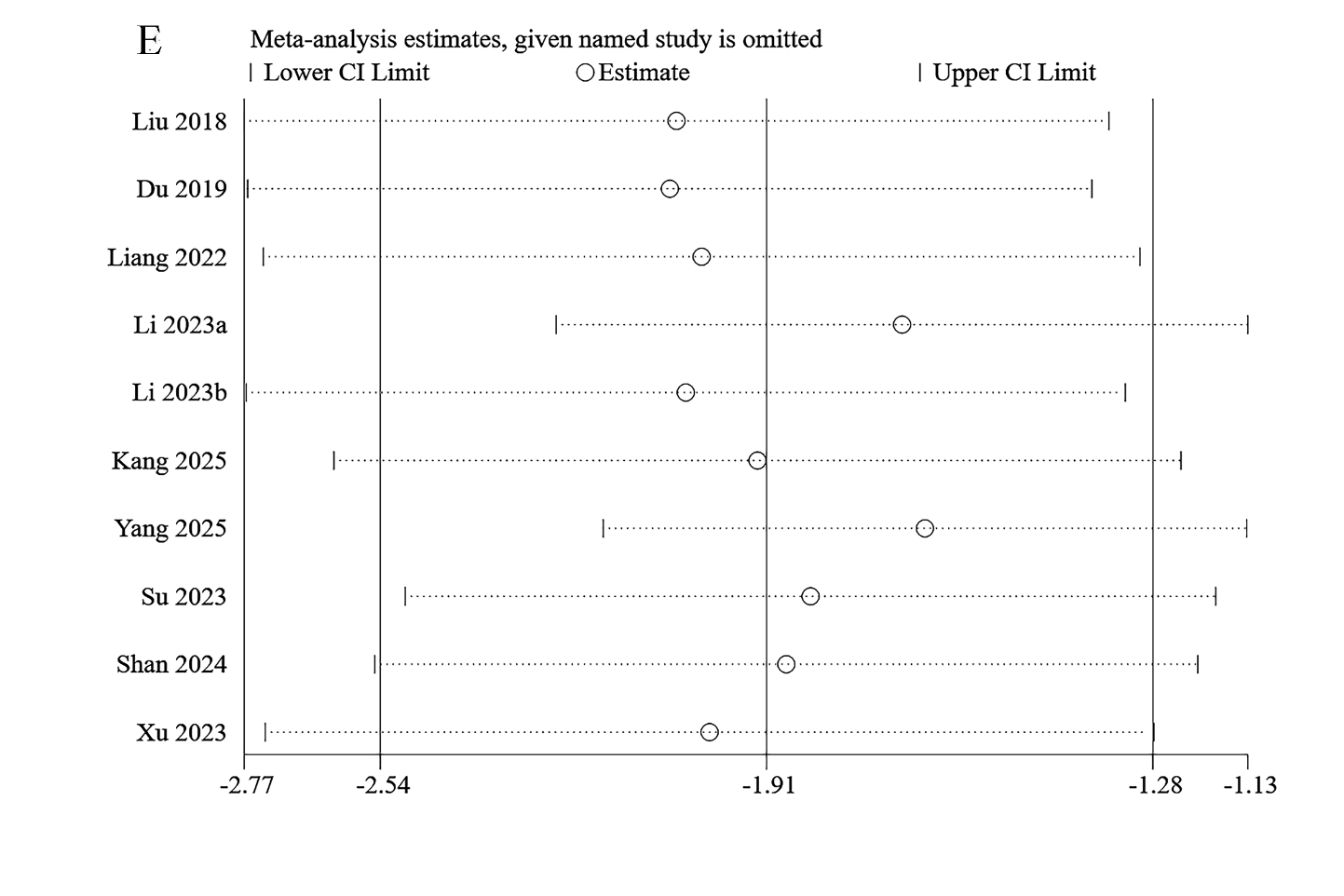


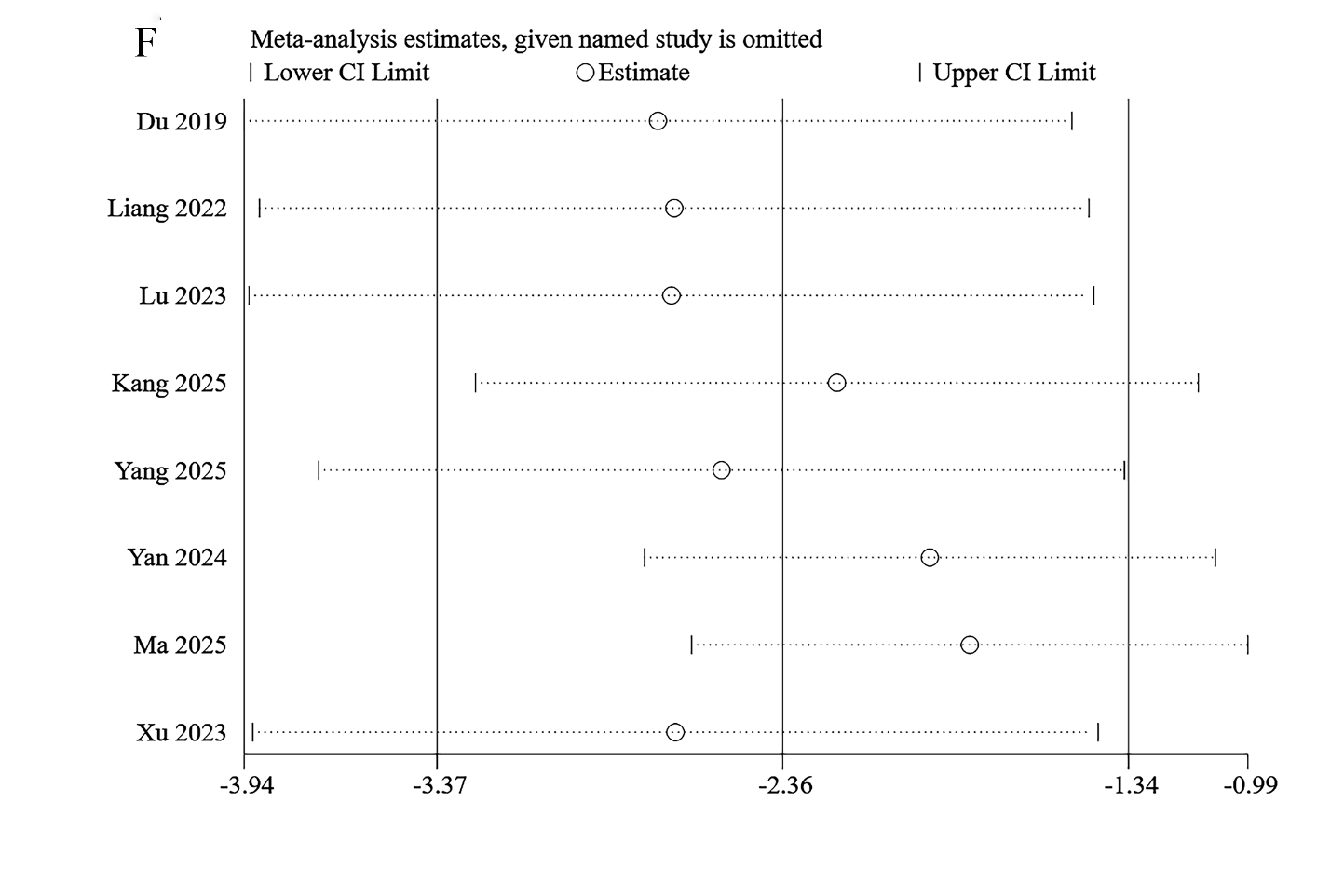


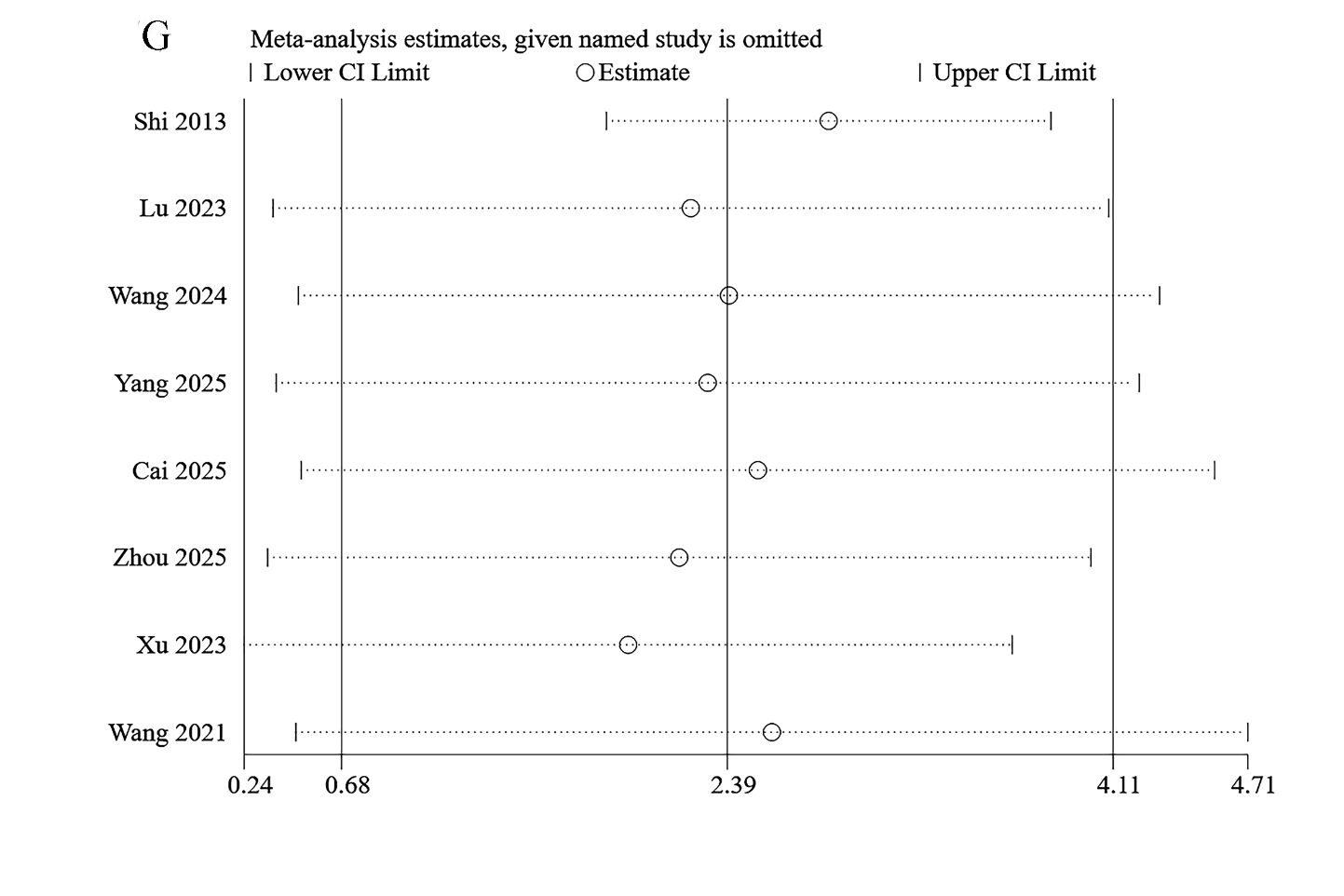


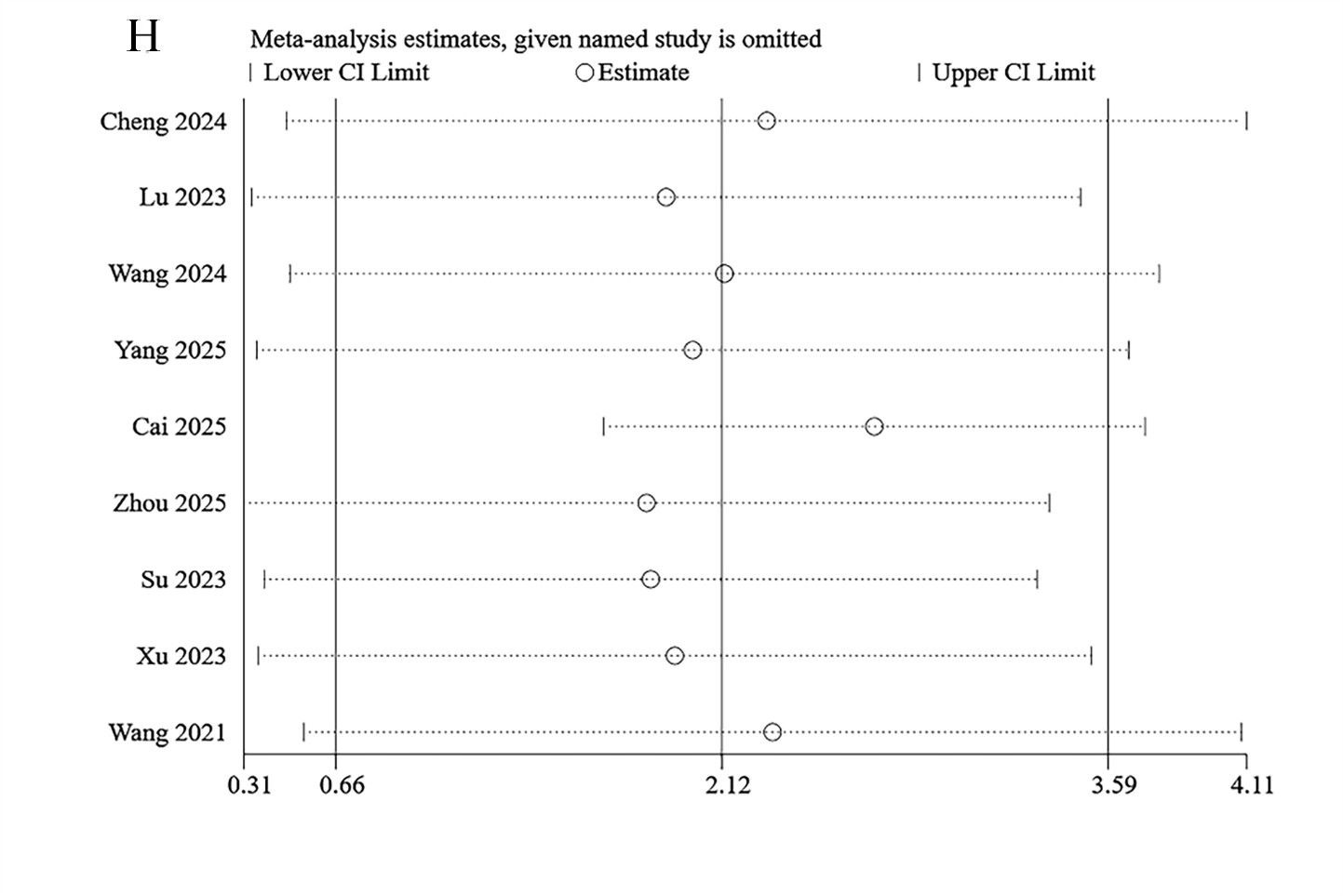


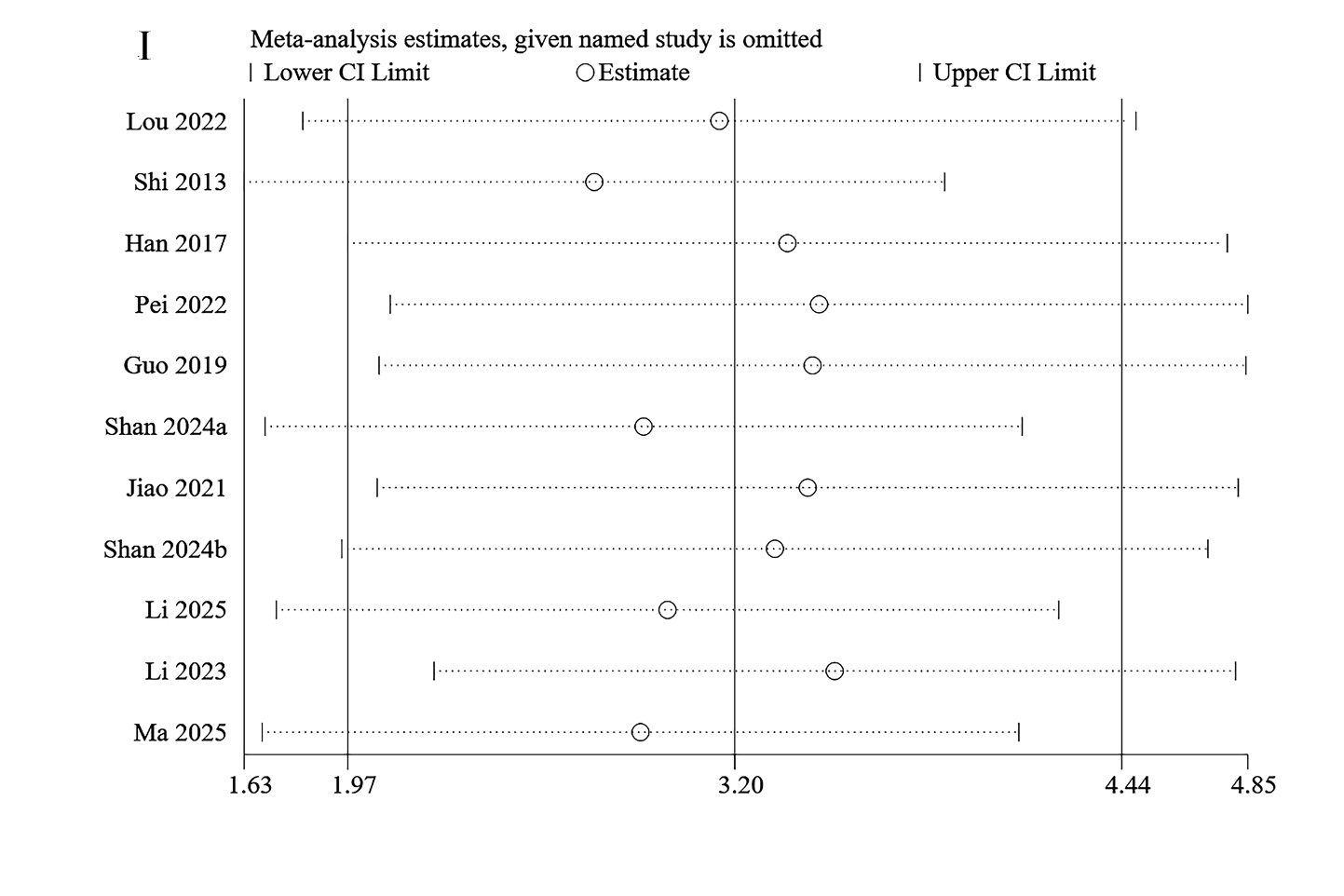


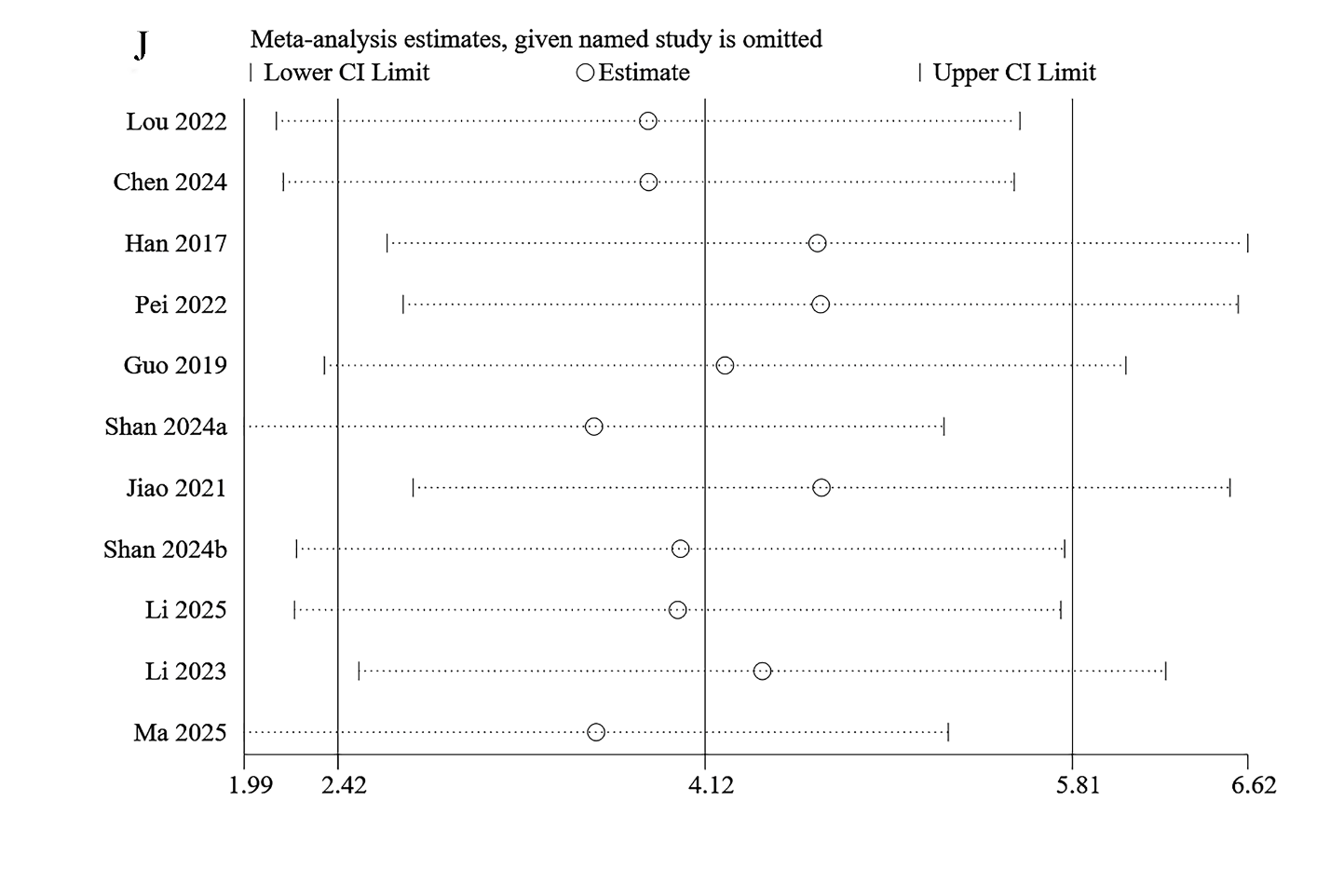


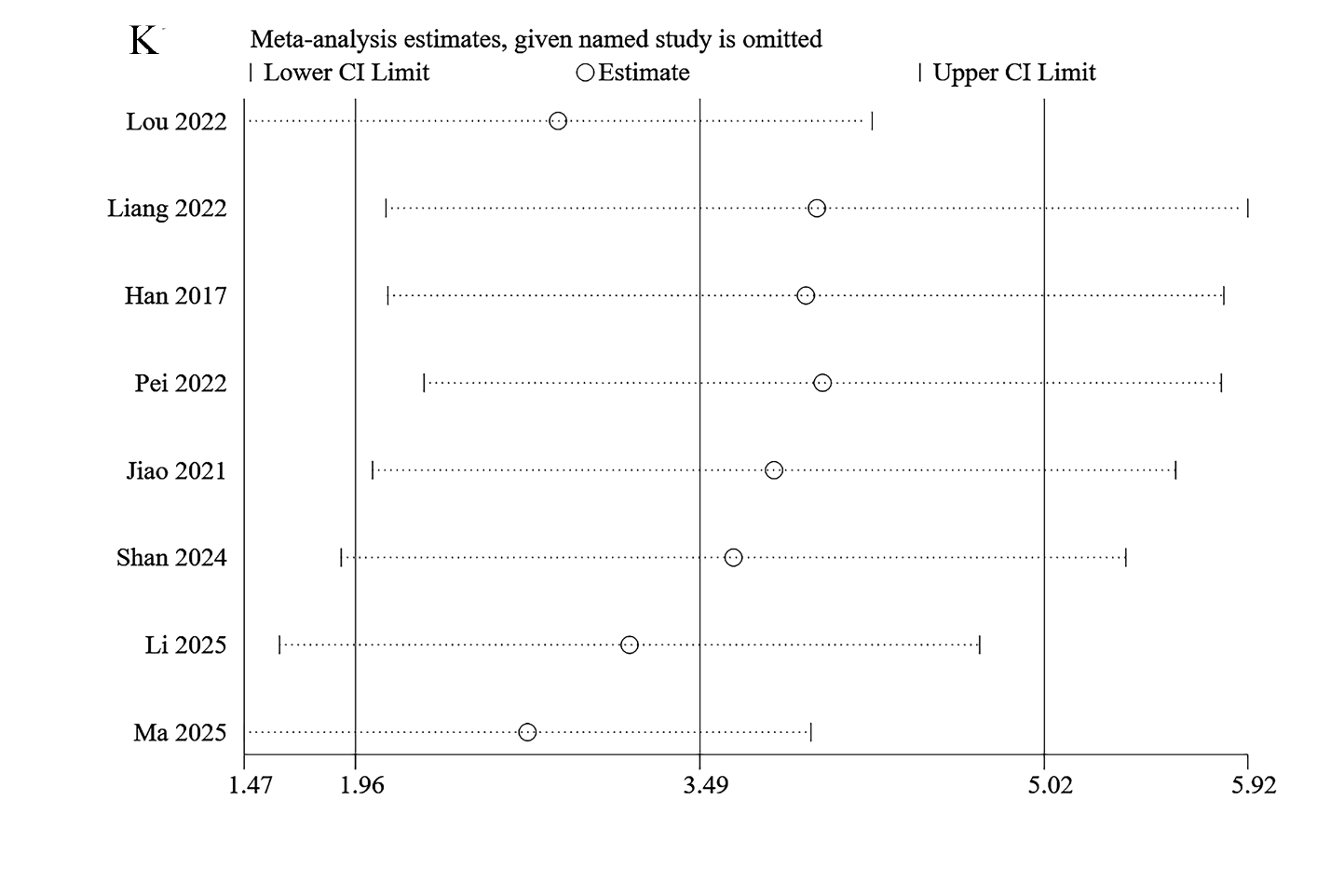


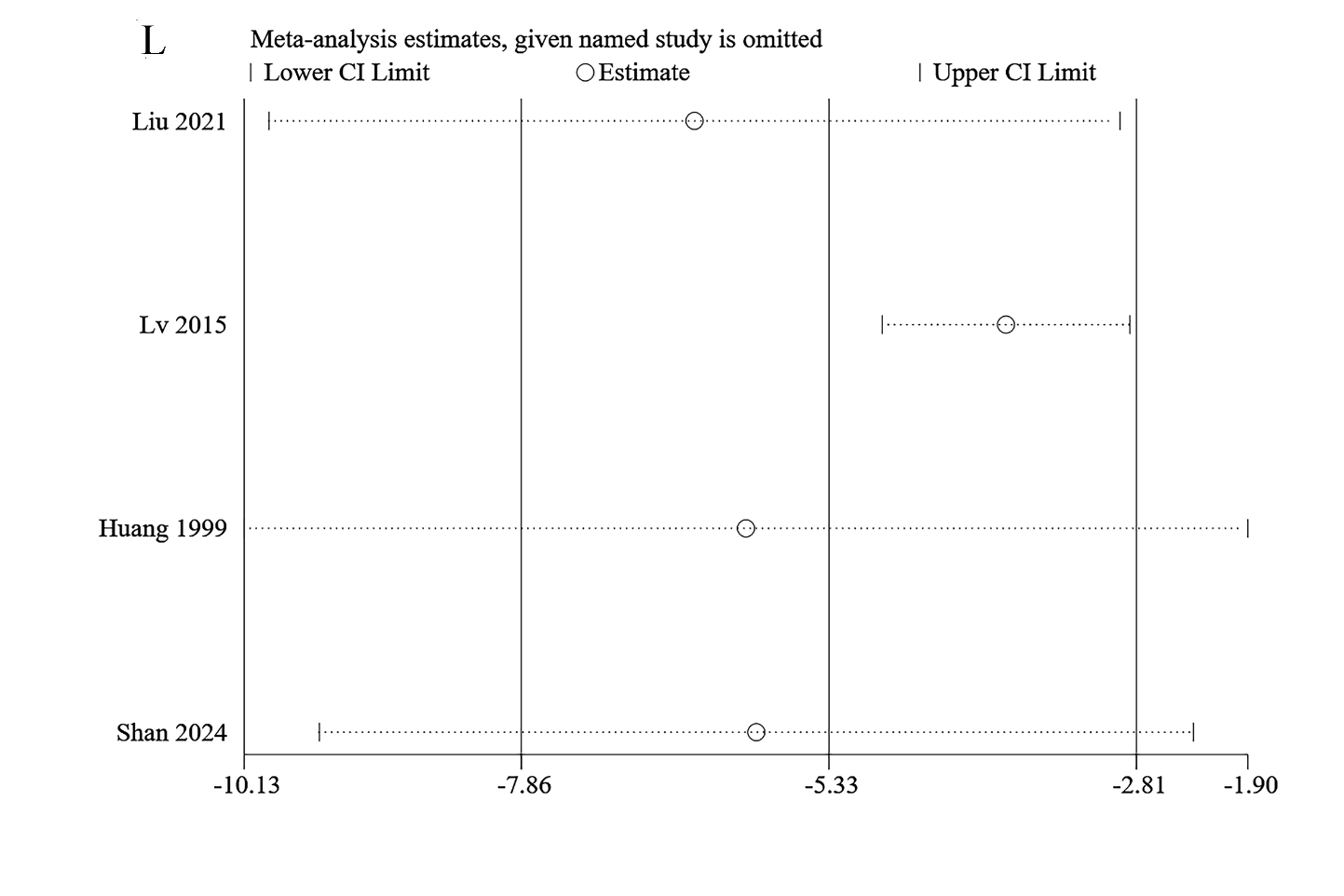


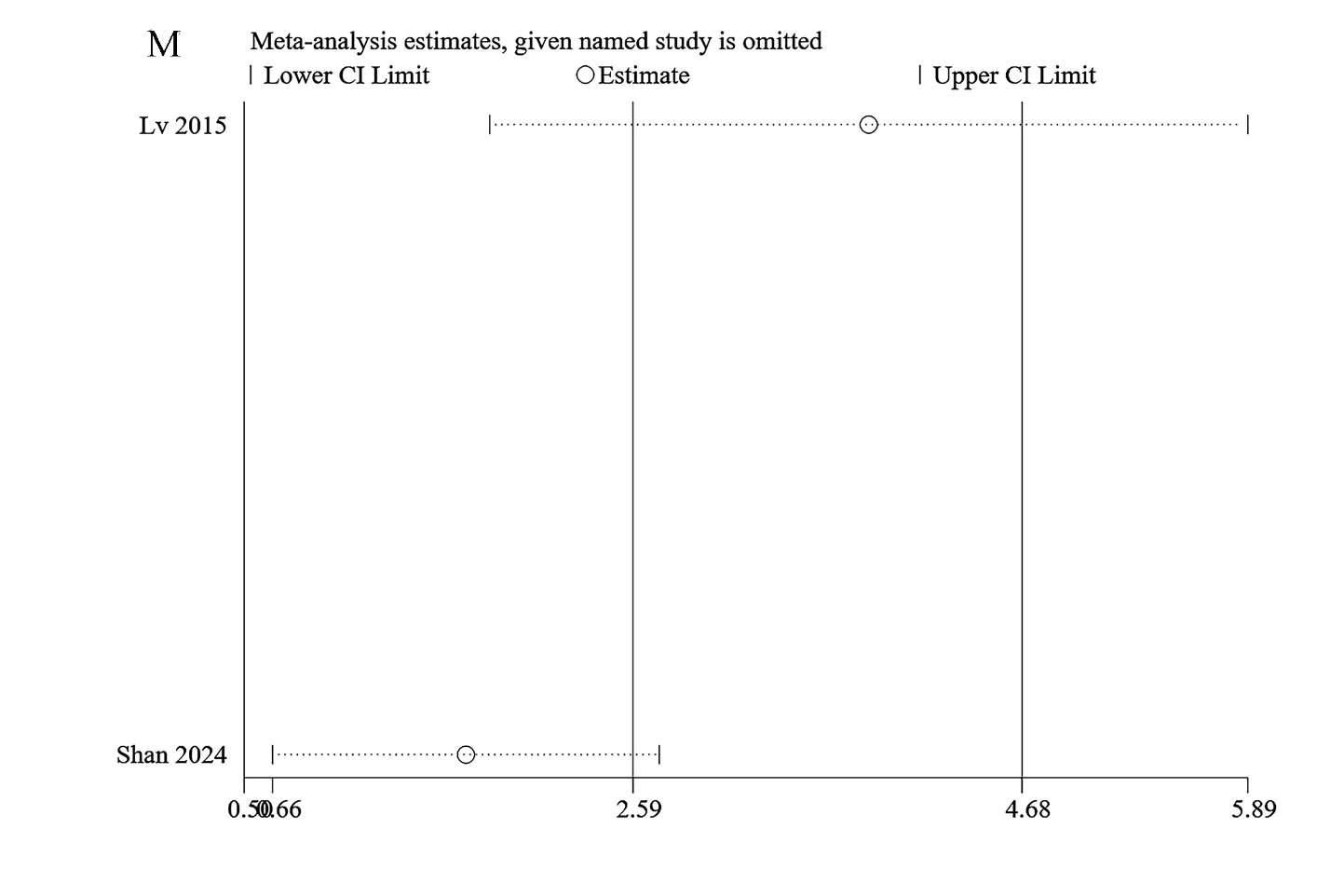


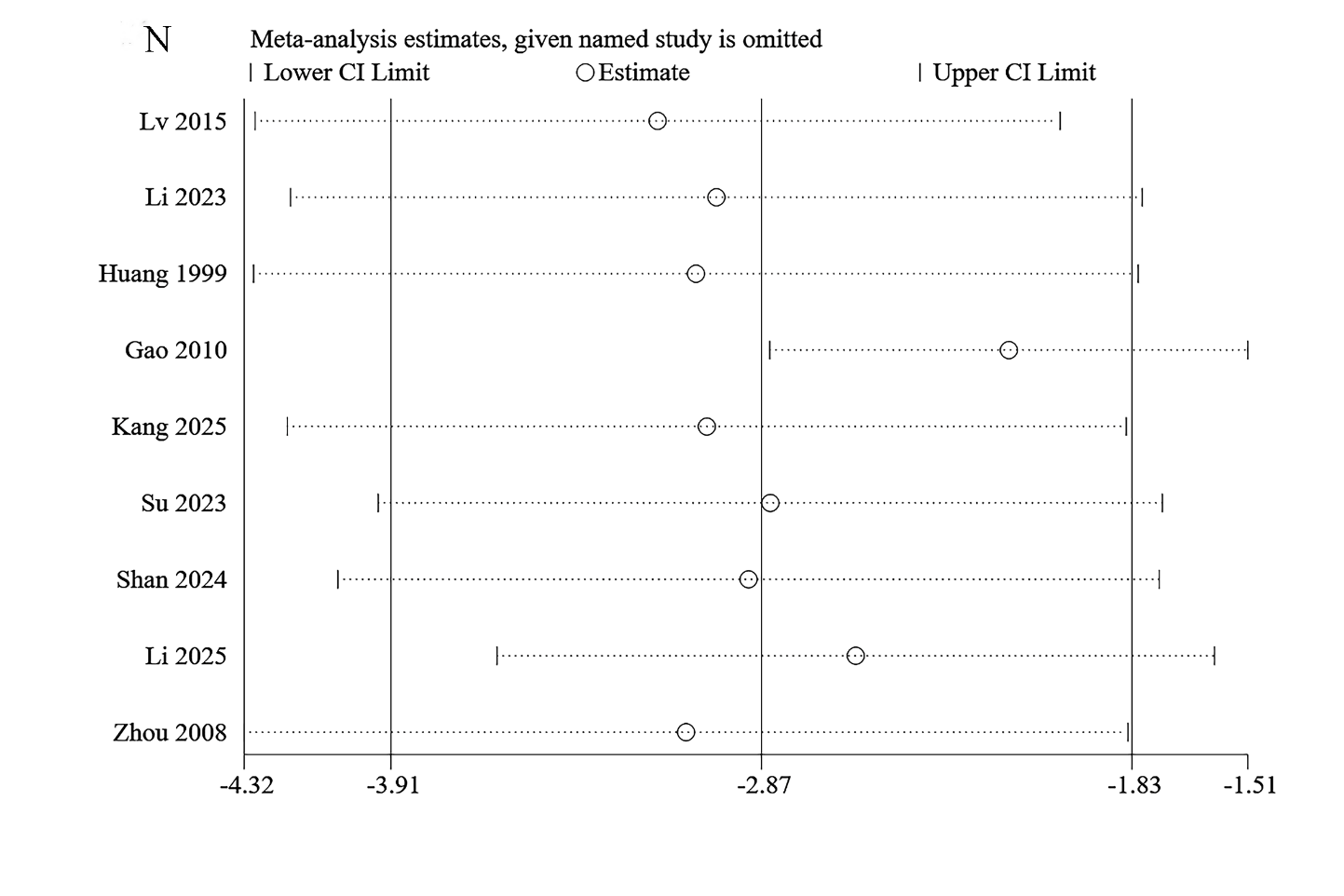


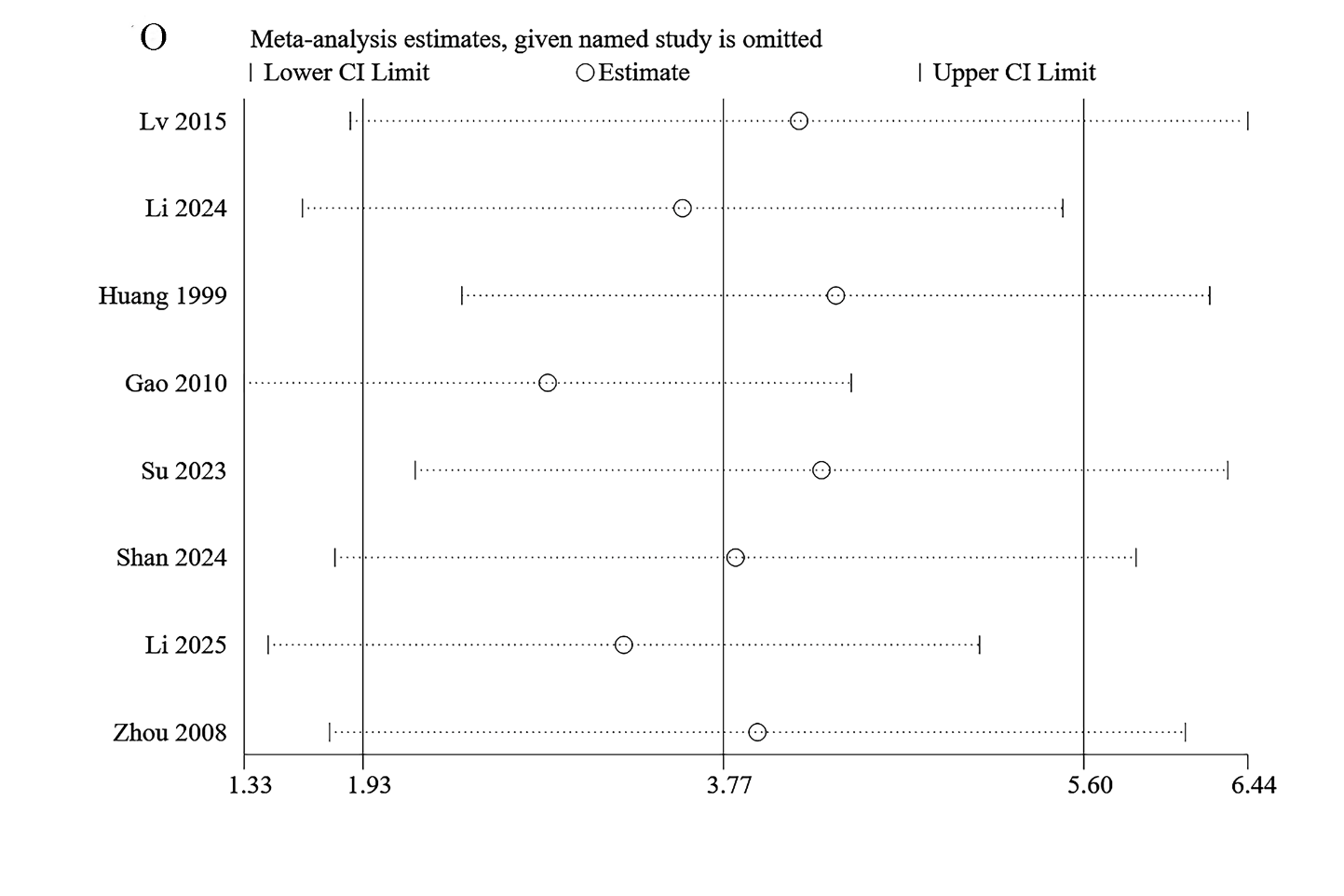


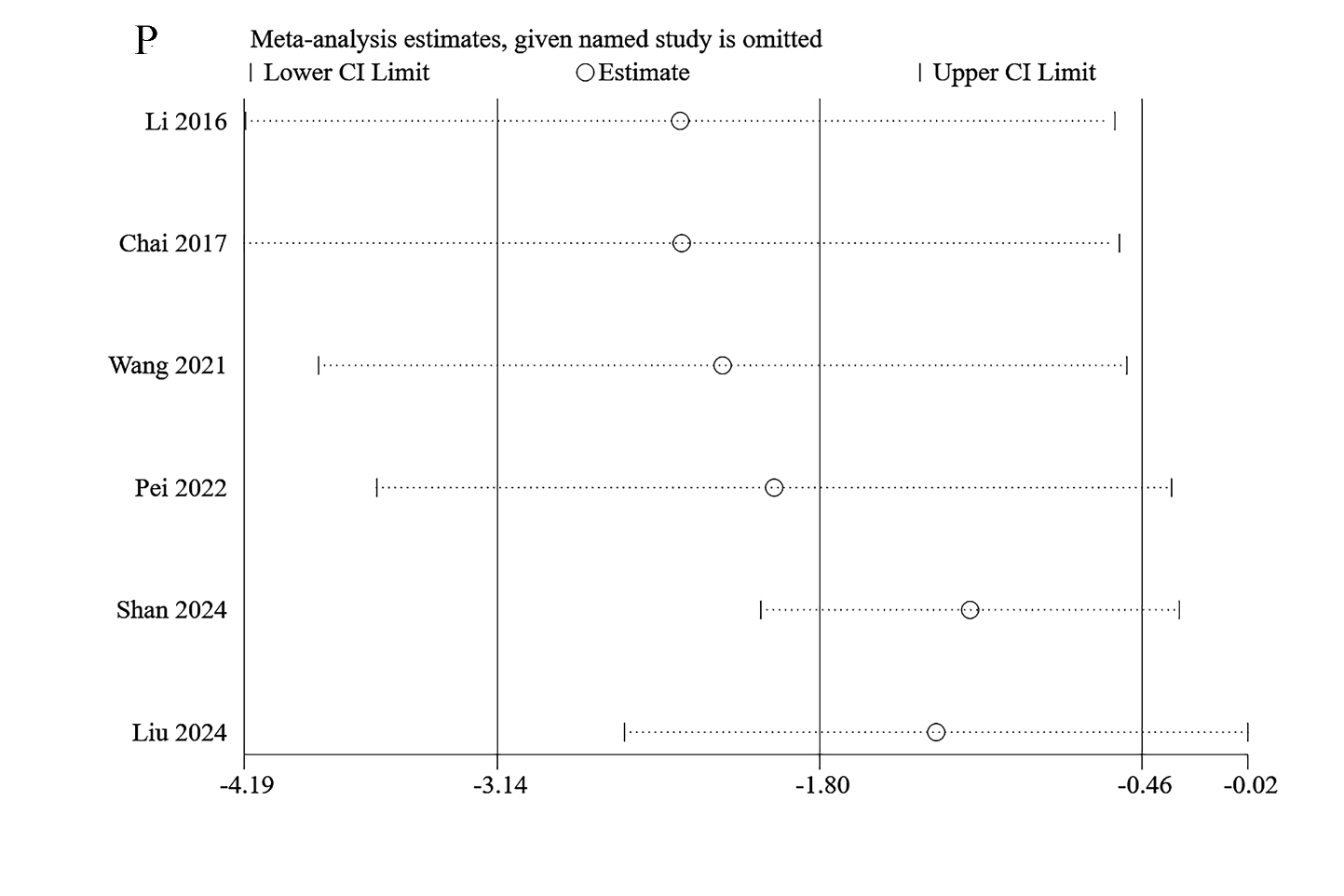


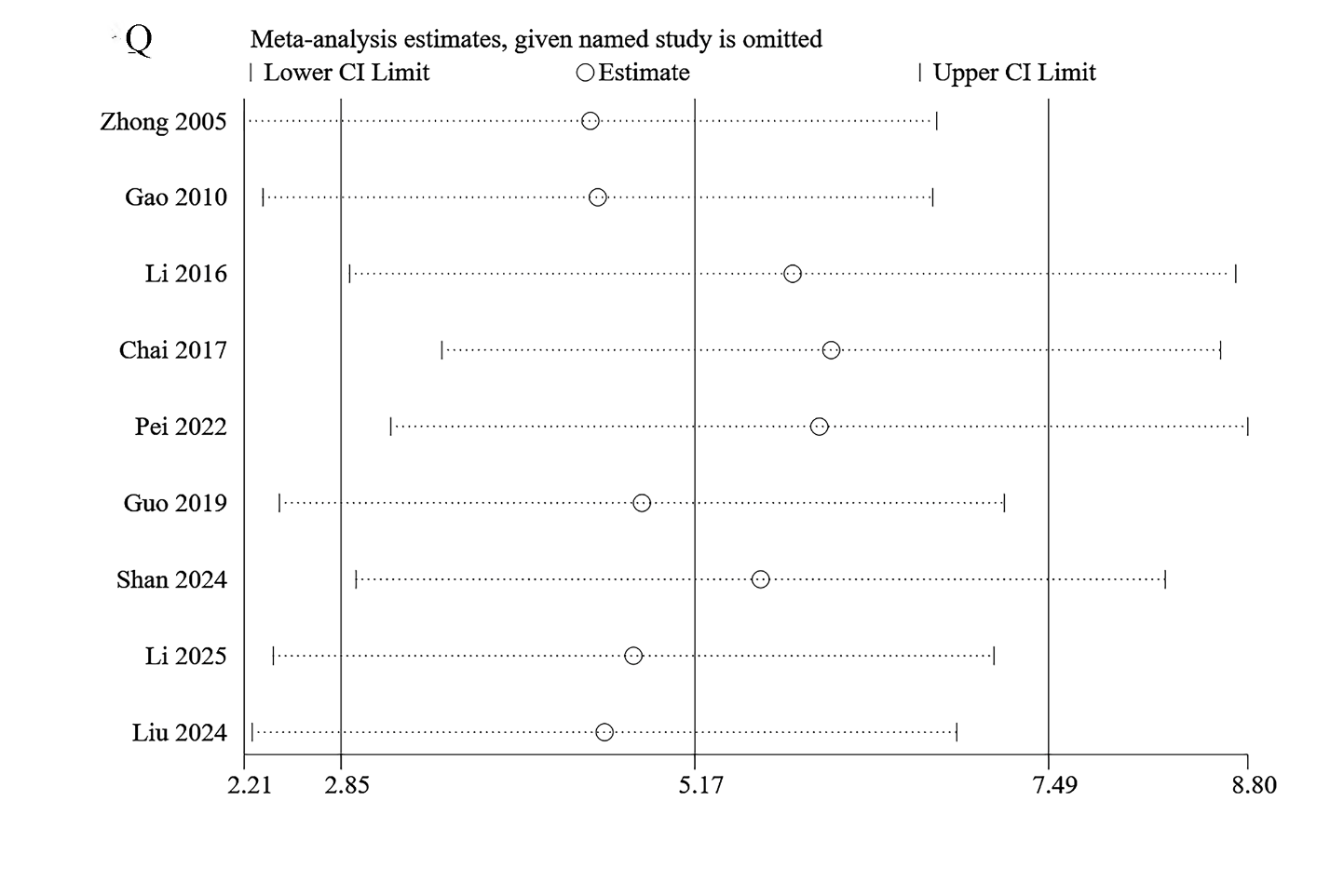


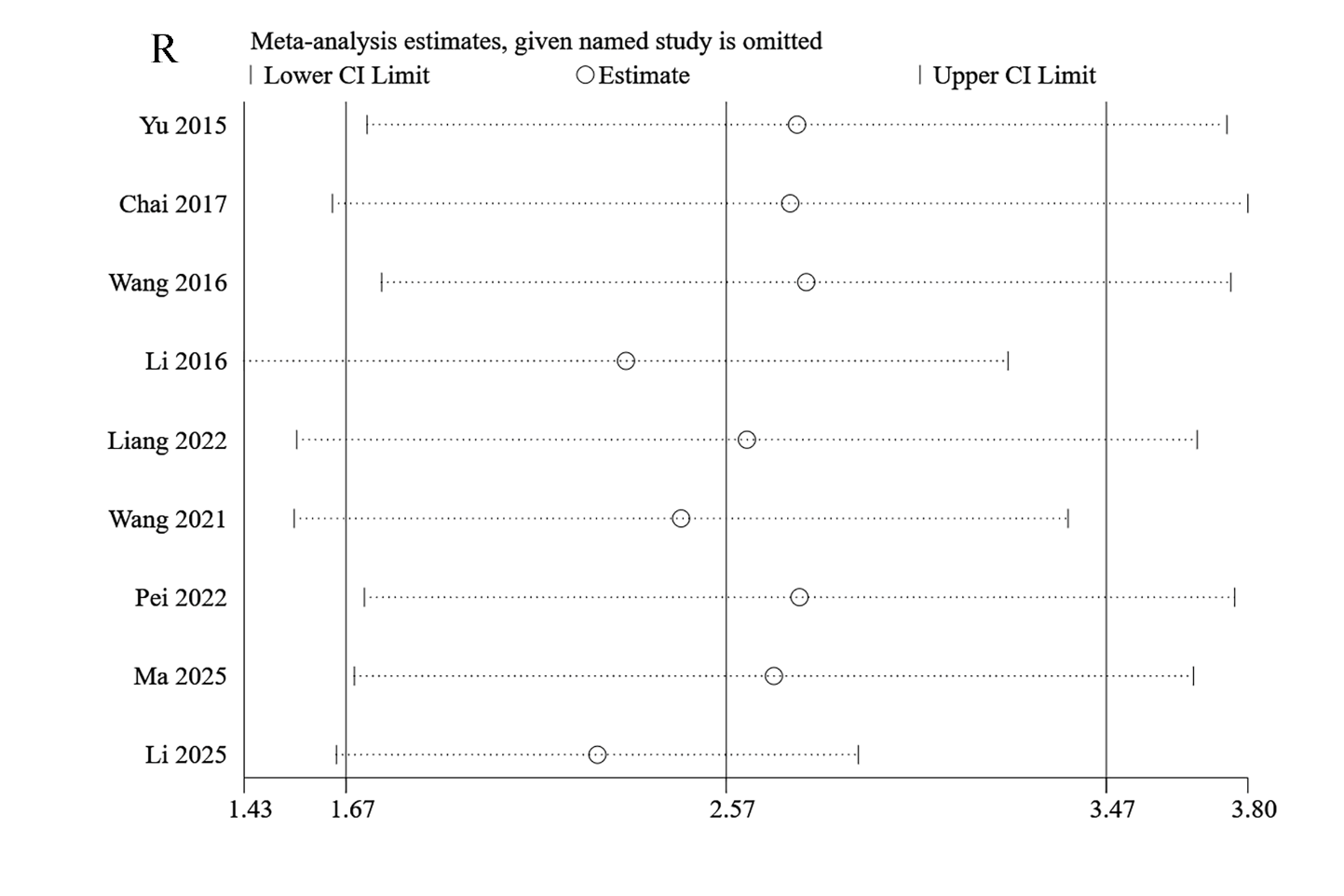


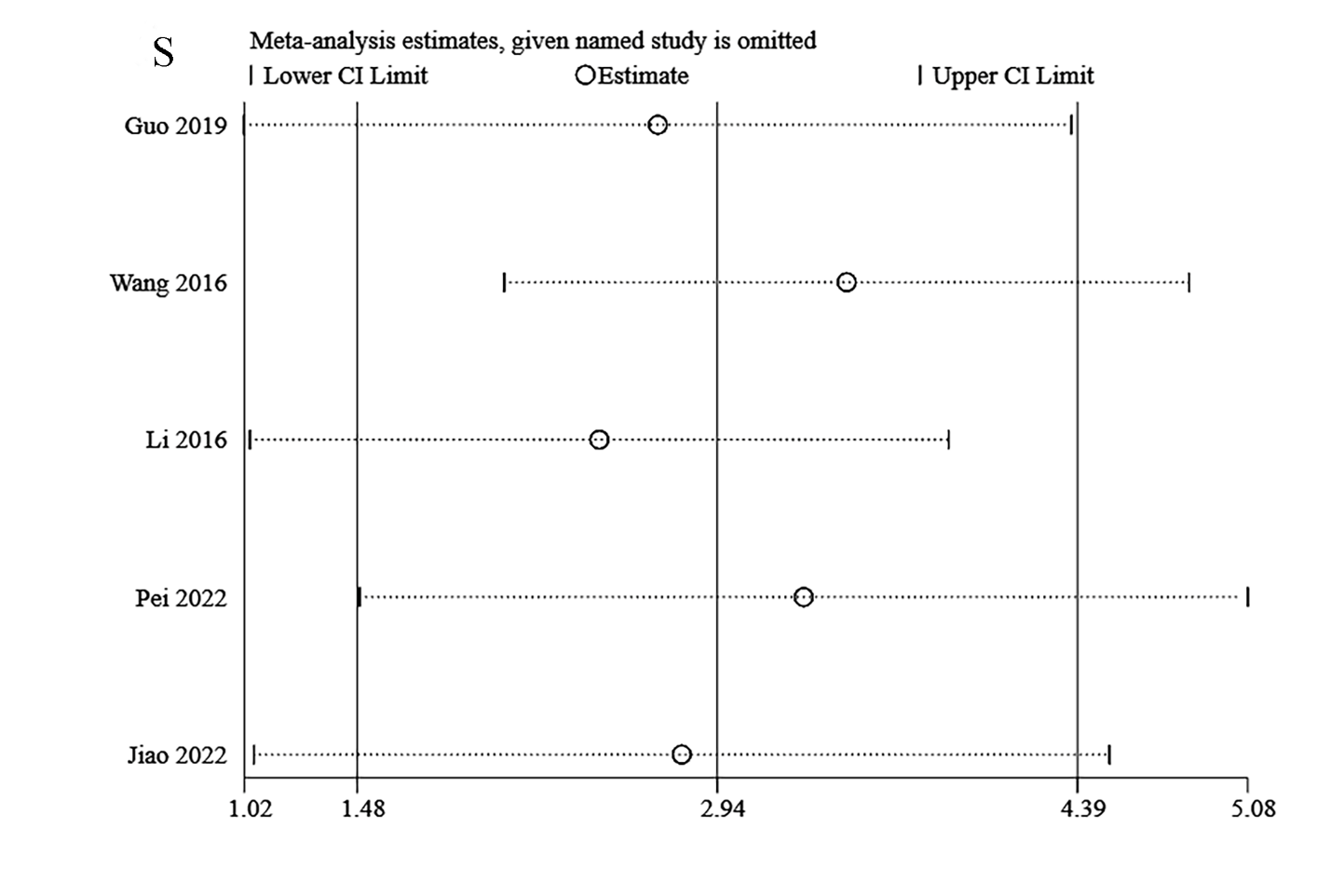


**Figure S1.** Sensitivity analysis. **(A)** escape latency of Morris water maze test; **(B)** entry frequency into the target quadrant of Morris water maze test; **(C)** time spent in the target quadrant of Morris water maze test; **(D)** crossing distance in the target quadrant of Morris water maze test; **(E)** relative identification index in the novel object recognition test; **(F)** spontaneous alternation in the Y-maze test; **(G)** tumor necrosis factor-α levels in the serum; **(H)** interleukin-1β levels in the serum; **(I)** tumor necrosis factor levels in the hippocampus; **(J)** interleukin-1β levels in the hippocampus; **(K)** interleukin-6 levels in the hippocampus; **(L)** superoxide dismutase activity in the serum; **(M)** malondialdehyde levels in the serum; **(N)** superoxide dismutase activity in the hippocampus; **(O)** malondialdehyde levels in the hippocampus; **(P)** acetylcholine content in the hippocampus; **(Q)** acetyl cholinesterase levels in the hippocampus; **(R)** Aβ, β-amyloid peptide content in the hippocampus; **(S)** tau content in the hippocampus.

## 3.2 Figure S2 Trim-and-fill analysis.


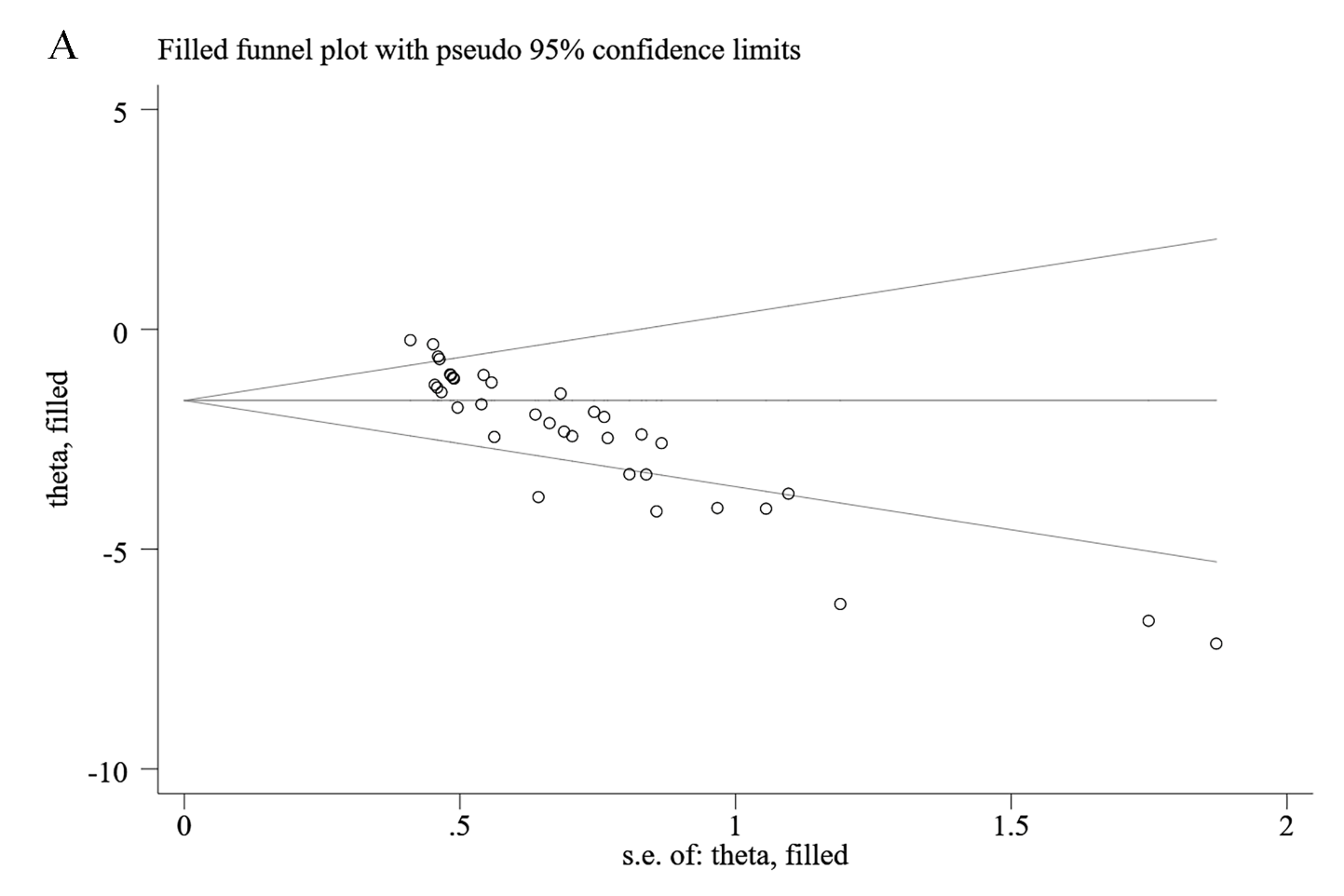


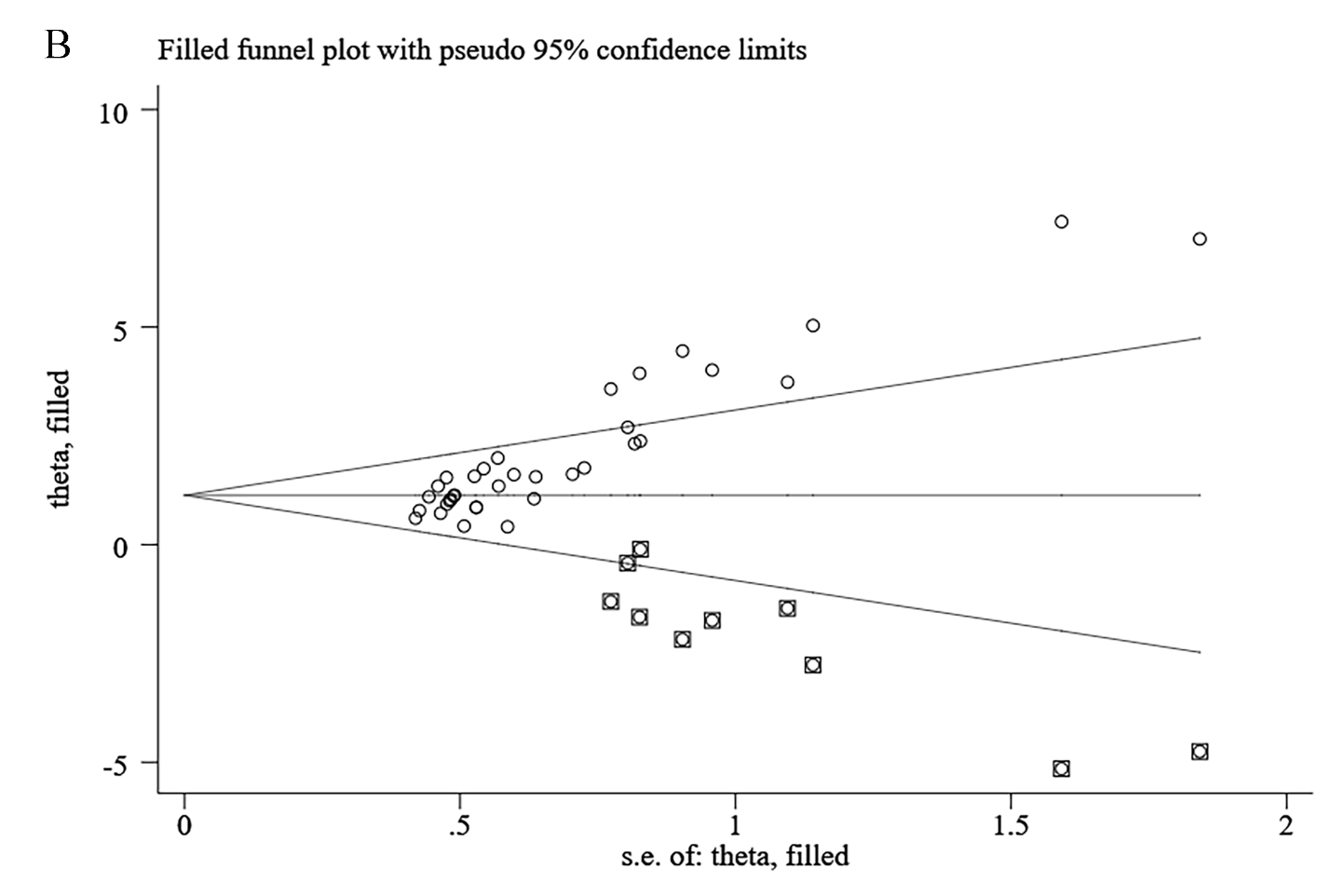


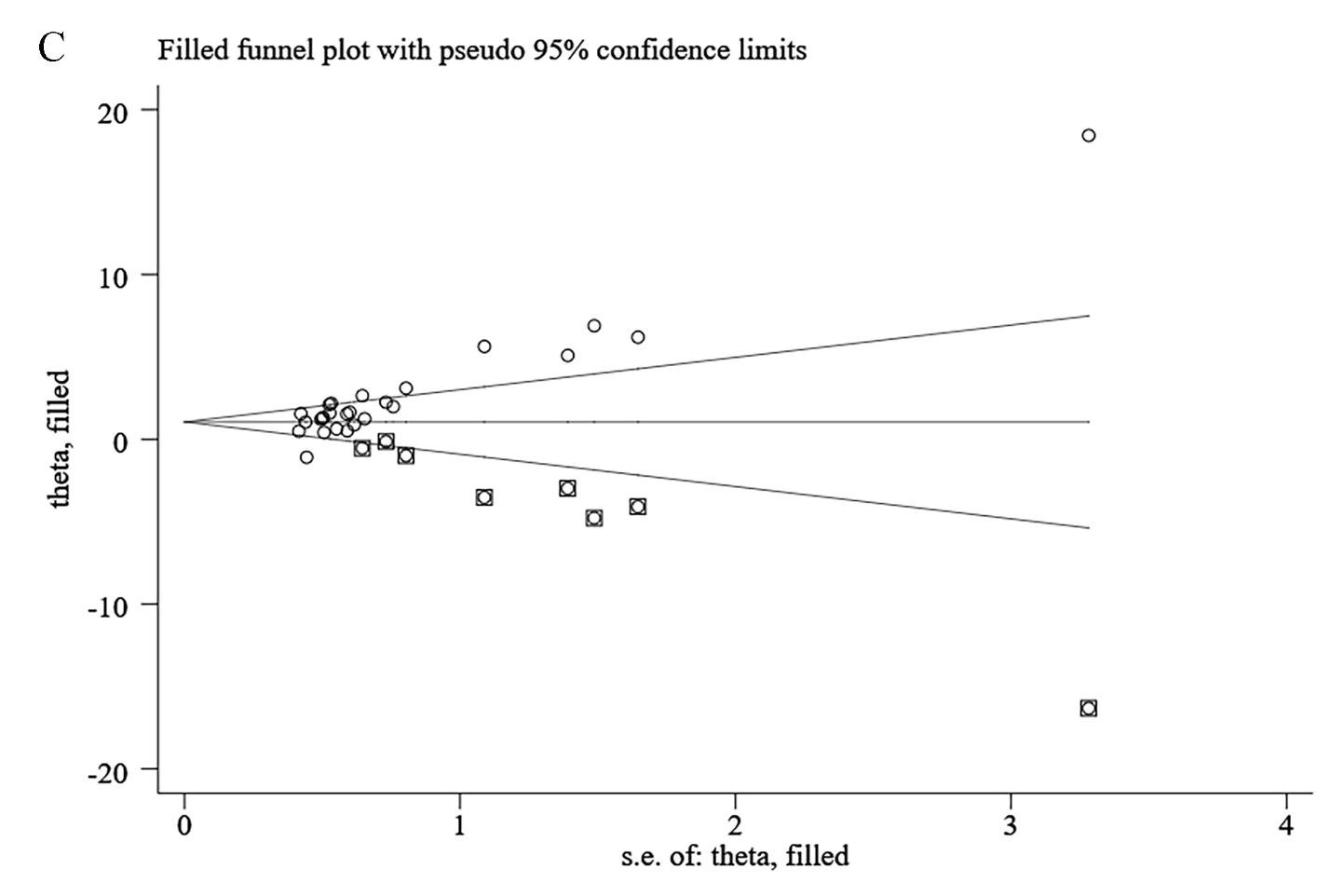


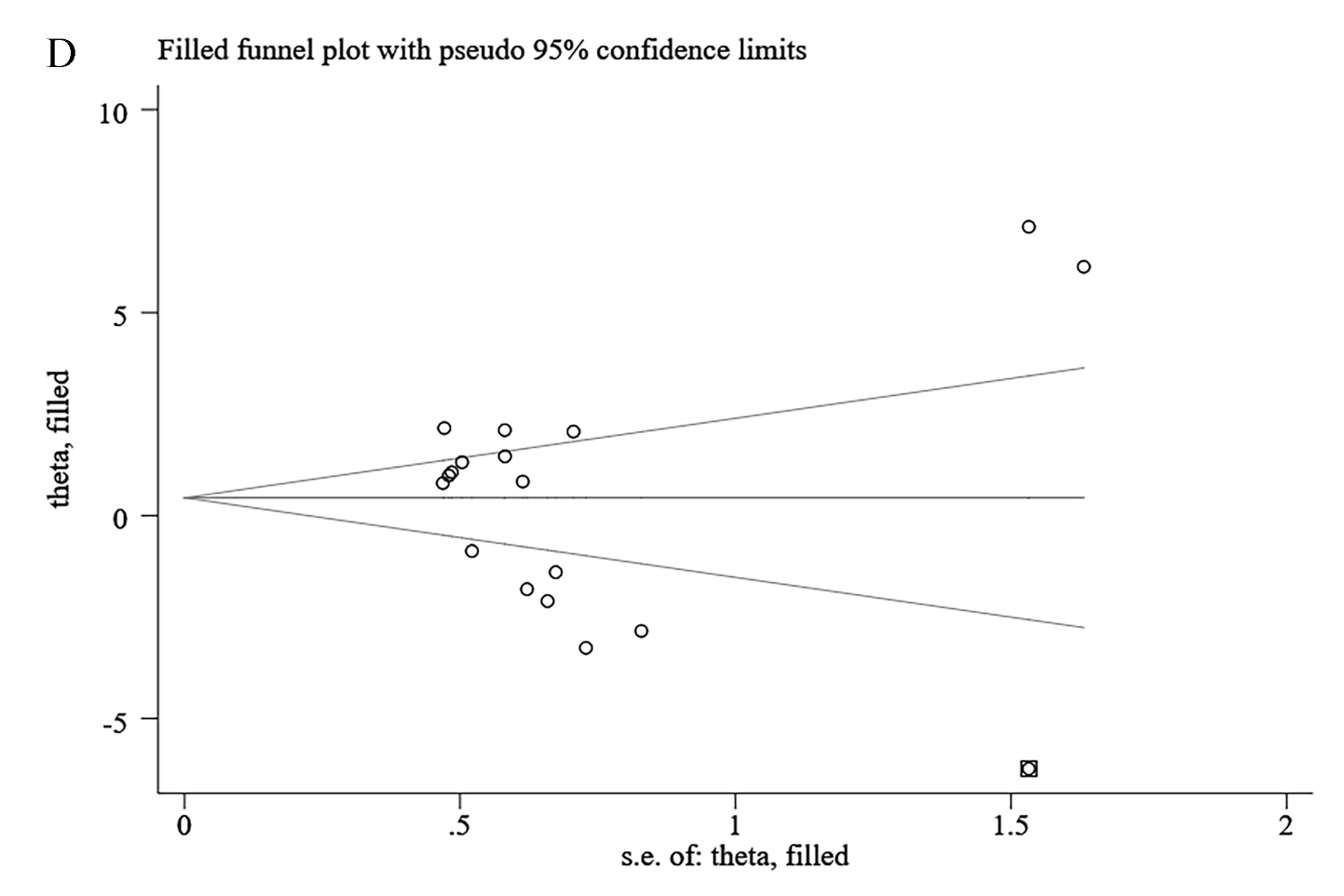


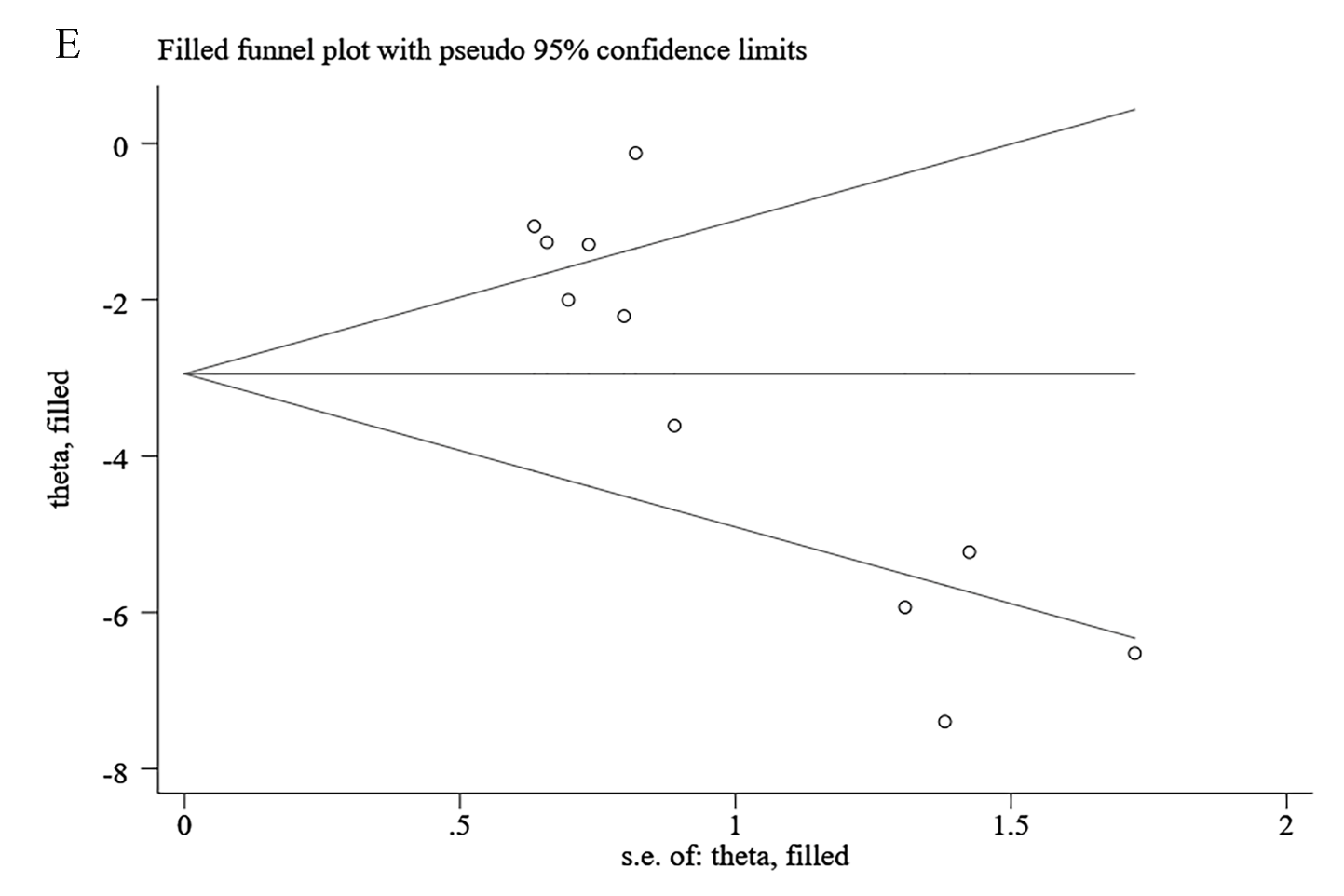


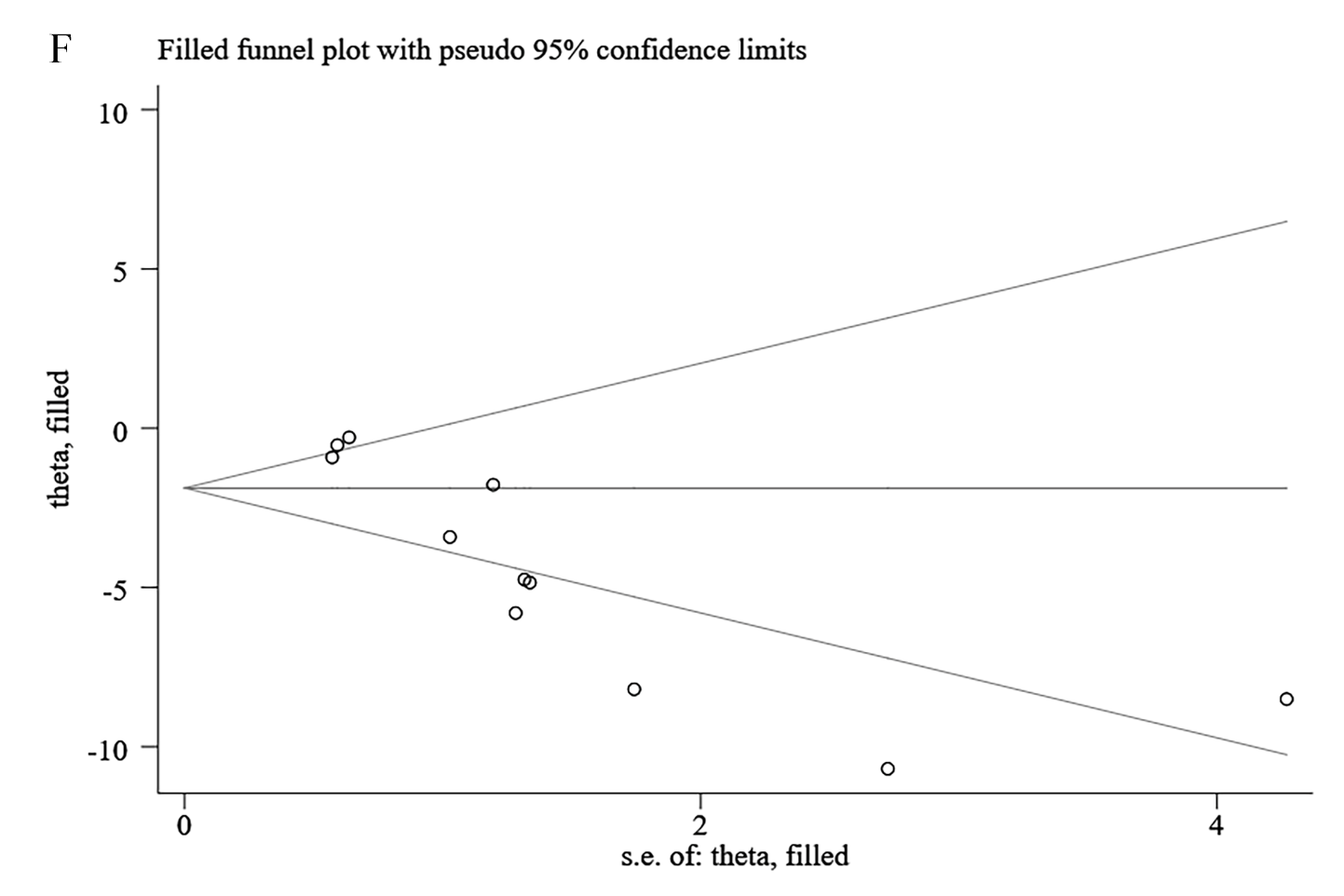


**Figure S2.** Trim-and-fill analysis. **(A)** Escape latency from the target quadrant; **(B)** Entry frequency into the target quadrant; **(C)** Time spent in the target quadrant; **(D)** Crossing distance in the target quadrant; **(E)** Tumor necrosis factor-α levels in the hippocampus; **(F)** interleukin-1β levels in the hippocampus.
